# Yiqi Huoxue Jiedu formula protects against sepsis-associated lung injury by modulating macrophage mitophagy and mtDNA-STING signaling

**DOI:** 10.1186/s13020-026-01475-0

**Published:** 2026-08-04

**Authors:** Shuqi Ma, Qiusha Pan, Jingnan Lin, Ruifeng Zeng, Xiaotu Xi, Ye Ye, Zhuoxu Gu, Kaiqiang Zhong, Suyi Yang, Lin Wang, Jun Li, Rui Chen

**Affiliations:** 1 The Second Clinical Medical School of Guangzhou University of Chinese Medicine, Guangzhou, 510405 China; 2 The Second Affiliated Hospital of Guangzhou University of Chinese Medicine & Guangdong Provincial Key Laboratory of Research On Emergency in Traditional Chinese Medicine, No. 111, Dade Road, Yuexiu District, Guangzhou, 510120 Guangdong China; 3 The First Clinical Medical School of Guangzhou University of Chinese Medicine, Guangzhou, 510405 China; 4 The First Affiliated Hospital of Guangzhou University of Chinese Medicine & Guangdong Clinical Research Academy of Chinese Medicine, 16 Jichang Road, Baiyun District, Guangzhou, 510405 Guangdong China; 5 Chinese Medicine Guangdong Laboratory, Zhuhai, 519000 China

**Keywords:** Yiqi Huoxue Jiedu formula, Sepsis-associated acute lung injury, Mitophagy, mtDNA-STING inflammatory axis

## Abstract

**Background:**

Yiqi Huoxue Jiedu Formula (YHJF) is a traditional Chinese medicine formula that has been used as an adjunctive therapy for sepsis for nearly two decades. Previous clinical studies showed that YHJF improves Sequential Organ Failure Assessment (SOFA) scores and modulates gut microbiota in elderly patients with pneumonia-associated sepsis. However, the mechanism by which YHJF protects against sepsis-associated acute lung injury (SALI) remains unclear.

**Methods:**

A murine SALI model was established by cecal ligation and puncture (CLP). Therapeutic effects were evaluated by histopathology, micro-CT, pulmonary function assessment, and ELISA. Mechanistic studies included proteomic analysis of lung tissues and LPS-stimulated MH-S macrophages, pharmacological modulation with Mdivi-1 and urolithin A (UA), macrophage-epithelial co-culture, HPLC fingerprinting, UPLC-HRMS, and molecular docking.

**Results:**

YHJF significantly improved 7-day survival and ameliorated lung injury, pulmonary edema, respiratory dysfunction, and systemic inflammation in mice with CLP-induced SALI. Proteomic profiling and subsequent functional assays suggested that enhanced mitophagy in macrophages represents a central protective mechanism. In vivo, YHJF increased autophagosome formation and PINK1/Parkin co-localization in BALF-derived alveolar macrophages. In vitro, YHJF restored mitochondrial homeostasis by activating PINK1/Parkin-dependent mitophagy in macrophages. This was accompanied by reduced cytoplasmic mtDNA leakage, downregulated cGAS expression, and suppression of the STING–TBK1–IRF3 pathway and subsequent type I interferon responses. Pharmacological inhibition of mitophagy with Mdivi-1 abolished these protective effects of YHJF, whereas activation with UA augmented them, demonstrating that mitophagy is necessary for YHJF-mediated protection. In a macrophage–epithelial co-culture system, YHJF-treated macrophages alleviated LPS-induced apoptosis in MLE-12 alveolar epithelial cells. Furthermore, chemical analysis integrated with molecular docking identified aloe-emodin, rhein, and genistein as candidate bioactive constituents of YHJF that likely contribute to its regulation of macrophage mitophagy.

**Conclusion:**

YHJF protects against SALI by restoring macrophage mitophagy and suppressing mtDNA-STING-mediated inflammatory signalling. These findings support YHJF as a potential therapeutic strategy for sepsis-associated lung injury.

**Graphical Abstract:**

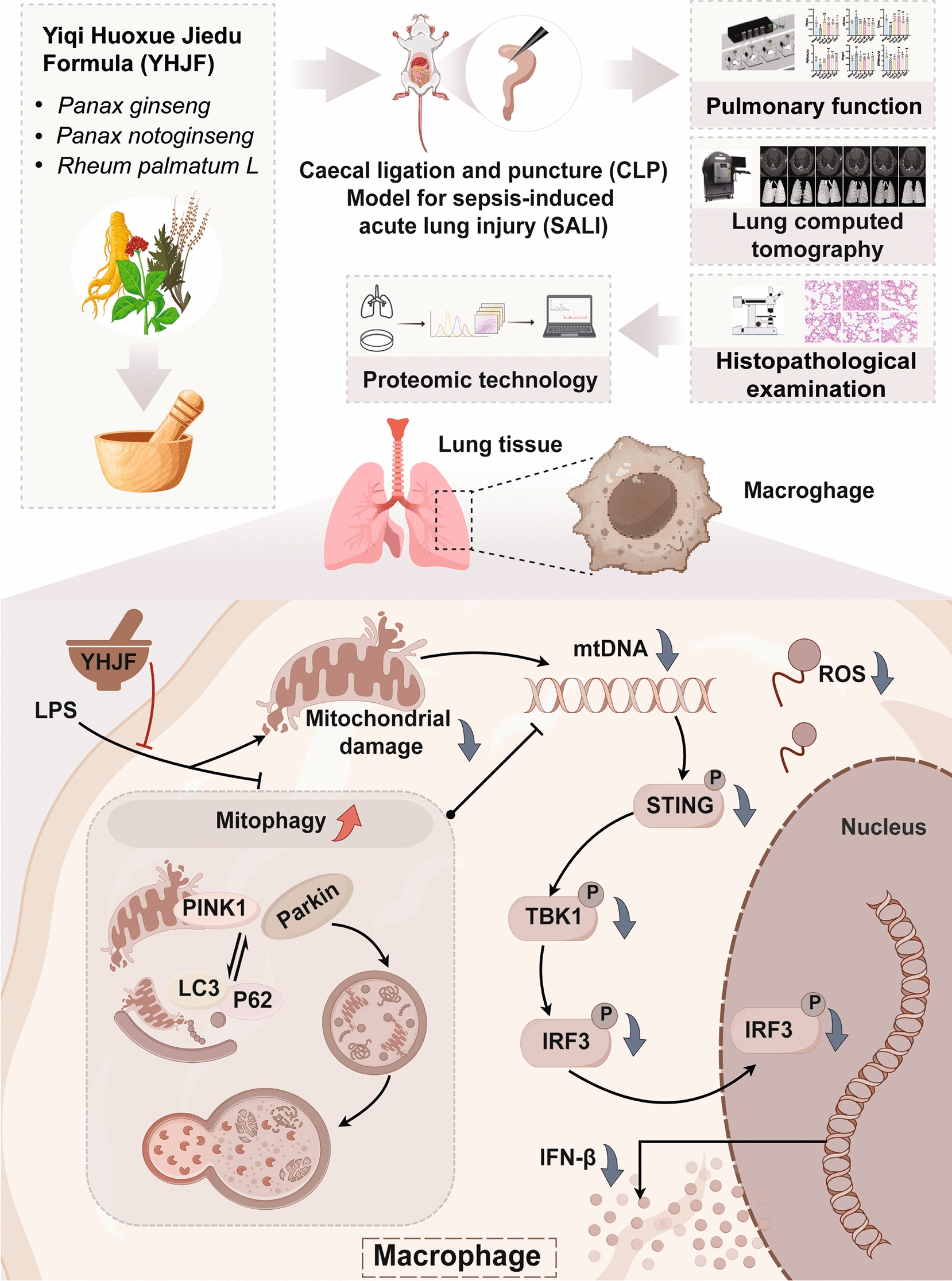

**Supplementary Information:**

The online version contains supplementary material available at 10.1186/s13020-026-01475-0.

## Introduction

Sepsis is a life-threatening organ dysfunction caused by a dysregulated host response to infection [1, 38]. Among its major complications, sepsis-associated acute lung injury (SALI) is particularly devastating, affecting 25–45% of septic patients and contributing substantially to mortality [21, 36]. Despite advances in supportive treatment, effective mechanism-based therapies for SALI remain limited. This therapeutic gap highlights the critical need to elucidate the actionable molecular pathways driving sepsis-induced lung damage.

Macrophages are pivotal mediators of tissue damage in SALI, driving pathological processes through mitochondrial dysfunction and excessive pro-inflammatory signaling [7, 45]. Under sustained pathogen- and cytokine-driven stress, impaired mitochondrial quality control promotes the accumulation of damaged mitochondria, excessive production of reactive oxygen species (ROS), and release of mitochondrial DNA (mtDNA), thereby amplifying innate immune signalling and compromising alveolar barrier integrity [24, 47]. PINK1/Parkin-mediated mitophagy serves as an essential protective mechanism by clearing dysfunctional mitochondria and limiting mtDNA-dependent inflammatory amplification [15, 27, 46]. However, in SALI, persistent pathogen and cytokine signaling inhibit the PINK1/Parkin pathway, impairing the clearance of damaged mitochondria [35]. When mitophagy is impaired, cytosolic mtDNA can activate the cGAS-STING pathway, further enhancing type I interferon responses and sustaining the inflammatory cascade [20, 28, 39]. Notably, impaired mitophagy not only exacerbates mtDNA-STING pathway overactivation but also maintains macrophages in a pro-inflammatory state [29]. Recent studies have demonstrated that enhancing mitophagy restores mitochondrial quality control and reduces mtDNA release, thereby alleviating lung inflammation [42, 49]. Thus, restoration of macrophage mitophagy may represent a promising strategy for suppressing mtDNA-STING-driven inflammation in SALI.

Traditional Chinese medicine (TCM) has been established as a viable complementary treatment for sepsis through extensive research, supported by extensive clinical and mechanistic evidence demonstrating its immunomodulatory potential [19, 27]. In TCM theory, sepsis is fundamentally associated with "deficiency of healthy qi (Zheng Xu) " and "excess of pathogenic factors (Xie Shi)", with deficiency, toxin, and stasis constituting key pathological features [22]. Under the guidance of National Master of Chinese Medicine Chen Shaohong, Professor Li Jun established the principle of reinforcing healthy qi throughout the course of sepsis management and developed Yiqi Huoxue Jiedu Formula (YHJF) by modifying the classical Renshen Dahuang Decoction from Bian Zheng Lu [17, 50], YHJF consists of Panax ginseng, Rhei Radix et Rhizoma, and Notoginseng Radix et Rhizoma, and has been widely used in clinical practice for nearly two decades. Our previous studies showed that YHJF improved SOFA scores, shortened ventilation duration and hospital stay, and modulated gut microbiota in patients with sepsis [5, 6, 16]. In addition, several constituent herbs and representative monomers of YHJF have been reported to exert anti-inflammatory or autophagy-regulating effects in sepsis-related injury [23, 51]. However, the mechanisms by which YHJF protects against SALI remain unclear.

In the present study, we employed in vivo and in vitro approaches to investigate the protective effects of YHJF against SALI and to elucidate its underlying mechanism. Using a cecal ligation and puncture (CLP) induced murine SALI model, LPS-stimulated MH-S macrophages, proteomic profiling, bidirectional pharmacological modulation of mitophagy, macrophage-epithelial co-culture, and chemical profiling, we tested the hypothesis that YHJF alleviates SALI by restoring macrophage mitochondrial homeostasis. Here we show that YHJF activates PINK1/Parkin-mediated mitophagy, limits mtDNA release, suppresses downstream STING-TBK1-IRF3 signalling, and attenuates alveolar epithelial cell apoptosis. These findings provide mechanistic insight into how YHJF modulates mitophagy and innate immune signaling in sepsis-associated lung injury, laying a clinical foundation for its future therapeutic use.

## Materials and methods

### Reagents and chemicals

The materials and instruments used in this study were as follows: RPMI-1640 (Gibco, C11875500BT); Fetal bovine serum (Gibco, A5669701); β-mercaptoethanol (ECODOP, ED-9153); Penicillin–streptomycin (Gibco, 15,140–122); Cell Counting Kit-8 solution (NCM, C6005); JC-1 Assay Kit (Beyotime, C2003S); ROS Assay Kit (Elabscience, E-BC-K138-F); ATP Assay Kit (Beyotime, S0026); Mdivi-1 (MedChemExpress, HY-15886); Urolithin A (MedChemExpress, HY-100599); Lpopolysaccharide (LPS) (Sigma, batch number L2630); BCA Protein Assay Kit (Thermo Scientific, batch number 23225); Protease inhibitor and phosphatase inhibitor (Roche, batch numbers 05892791001 and 04906837001, respectively); Hematoxylin and eosin (HE) staining kit (Leigen, batch number DH0006); Enzyme-linked immunosorbent assay (ELISA) kits for IL-6, TNF-α, and IL-1β (Cusabio, batch numbers E04639m, E04741m, and E08054m, respectively); Annexin V-FITC/PI Apoptosis Detection Kit (KGA1102); SteadyPure Universal RNA Extraction Kit, Evo M-MLV Reverse Transcription Premix Kit, and SYBR Green Pro Taq HS Premixed qRT-PCR Kit (AG, batch numbers AG21017, AG11728, and AG11718, respectively); HRP-conjugated goat anti-rabbit and anti-mouse secondary antibodies (CST, 7074S and 7076S); p62 and cGAS (CST, 23214, 31659 T); Phosphorylated STING (p-STING), Phosphorylated TANK-binding kinase 1 (p-TBK1); TBK1, Phosphorylated interferon regulatory factor 3 (p-IRF3), and mitochondrial transcription factor antibodies (Affinity, batch numbers AF7416, AF8109, AF8190, AF2436, and AF0531, respectively); Translocase of the outer membrane 20 (TOM20) and STING antibodies (Proteintech, batch numbers 11802-1-AP and 19851-1-AP, respectively). Transwell insert (0.4 μm; Corning),

Multifunctional microplate reader (M1000pro, Tecan); Electrophoresis apparatus (041BR92965, Bio-Rad); Protein blot imaging system (ChemiDoc MP, Bio-Rad); Gradient polymerase chain reaction (PCR) instrument (T100, Bio-Rad); Fluorescent quantitative PCR instrument (ViiA7, ABI); Automated inverted fluorescence microscope system (Ti2-E, Nikon).

### Preparation of extract and containing serum of YHJF

YHJF comprises 30 g of Panax ginseng (Renshen), 18 g of Notoginseng (Sanqi) (purchased from Lingnan Chinese Medicine and Drinks Company Limited, 2404001, 2403002), and 6 g of Rhei Radixet Rhizoma (Dahuang) (purchased from Sinopharm Group Feng Dai Sexy Herbal Drinking Tablets Co, C12308141). All Chinese herbal ingredients are provided by Guangdong Provincial Hospital of Traditional Chinese Medicine. These three herbs were soaked for 30 min and then decocted twice. The resulting decoctions were condensed using a vacuum concentrator and stored at −80 °C for further use.

YHJF-containing serum was prepared using male Sprague–Dawley rats randomly assigned to blank serum and YHJF-treated serum groups. Rats in the YHJF-treated group received three oral gavages of YHJF at the clinically equivalent dose, whereas rats in the blank serum group received an equal volume of saline. Serum was collected, heat-inactivated at 56 °C for 30 min, filtered, and stored for subsequent in vitro experiments. Blank serum was used as the corresponding vehicle control in all serum-based cell experiments.

### Chemical characterization and quality control of YHJF

The chemical profile of YHJF was characterized by HPLC fingerprinting and UHPLC-Q-Exactive Orbitrap HRMS. For comprehensive constituent identification, the extract was further analyzed by UHPLC-Q-Exactive Orbitrap HRMS (Acquity UPLC BEH C18, 100 × 2.1 mm, 1.7 μm; 0.1% formic acid in water/methanol gradient at 0.3 mL/min). MS detection was performed in both positive and negative HESI modes, and compounds were identified based on accurate mass (< 10 ppm error), retention behavior, MS/MS fragmentation, and comparison with reference standards, published data, and databases (PubChem, MassBank, OTCML). All reference standards were purchased from TargetMol Chemicals Inc. (Shanghai, China).

For HPLC analysis, the extract (0.2 g/mL crude drug equivalent in 70% methanol, filtered through 0.22 μm) was separated on a PerkinElmer Quasar AQ C18 column (250 × 4.6 mm, 5 μm; 40 °C) using a water-acetonitrile gradient (1.0 mL/min, 20 μL injection, 210 nm detection). Reference standards—including ginsenoside Rg1, Re, and Rb1 (for Panax ginseng), notoginsenoside R1 (for Panax notoginseng), and aloe-emodin, rhein, and emodin (for Rheum palmatum)—were used for fingerprint validation per the Chinese Pharmacopoeia (2025 edition).

### Animal experiments

All animal experiments were performed in accordance with the ARRIVE guidelines and were approved by the Guangdong Provincial Traditional Chinese Medicine Ethics Committee (Approval No. 2024085). Female BALB/c mice (6–8 weeks old) were obtained from the Animal Experiment Center of Guangzhou University of Chinese Medicine [Certificate No. SCXK (Yue) 2023–0068] and acclimatized for one week before use. Mice were stratified by body weight and randomly assigned to six groups: Sham, CLP, YHJF-L (3.51 g/kg/day), YHJF-M (7.02 g/kg/day), YHJF-H (14.04 g/kg/day), and DEX (5 mg/kg/day). For the survival study (n = 15 per group), treatments began immediately after CLP induction and continued for 7 consecutive days. For the pharmacological study (n = 6 per group), intragastric administration started 72 h prior to CLP induction. In both experiments, survival (for the survival study) and relevant pharmacological outcomes were systematically monitored. The medium dose of YHJF in mice (7.02 g/kg/day) is clinically equivalent to the dose used in a 70 kg adult. The dexamethasone (DEX) positive control group received 5 mg/kg, while the Sham group and CLP received an equal volume of saline solution. All groups were administered the treatment twice daily simultaneously.

### CLP model preparation

A murine SALI model was established by cecal ligation and puncture (CLP). After induction of anesthesia using isoflurane, the mice were positioned in a supine orientation. A midline abdominal incision measuring 1 cm was made to access the cecum, which was subsequently ligated 1 cm from the apex and punctured twice with an 18-gauge needle to allow fecal extrusion. Following the surgical procedure, saline was administered subcutaneously at a volume of 0.5 mL per 10 g body weight to facilitate fluid resuscitation. Tissue and serum samples were collected 24 h after the model was established.

### Histopathological staining

Mouse right upper lung lobes were fixed in 4% paraformaldehyde, embedded in paraffin, and sectioned at 4 μm thickness. Sections were stained with hematoxylin and eosin. Lung pathology was evaluated by light microscopy (Axio Imager M2, ZEISS) using the Murray Lung Injury Scoring [33].

### Micro-CT analysis of mouse lungs

At 24 h post-CLP, lung injury was assessed by micro-CT imaging. Mice were anesthetized with 3% isoflurane in air and maintained on a 37 °C heated bed during scanning. The Skyscan 1276 system (Bruker, Germany) was employed with the following parameters: 70 kVp, 80 µA, 4 min scan time, 36 mm field of view, and 72.0 µm pixel size. Image analysis and 3D reconstruction were performed using CTan and CTvox software (Bruker).

### Lung function assessment, BALF collection, and lung W/D ratio

Mice were individually placed in the EMKA-WBP-RT4 whole-body plethysmography system (EMMS). After respiratory patterns stabilized, non-invasive lung function measurements were recorded continuously for 10 min.

To collect bronchoalveolar lavage fluid (BALF), the lungs were gently lavaged four times with ice-cold PBS. Each lavage consisted of 0.4 mL for the first instillation, followed by 0.3 mL for each of the three subsequent instillations. After each instillation, the lavage fluid was slowly withdrawn and collected. Centrifuge the collected BALF and use the BCA protein assay kit to determine the protein concentration of the supernatant.

For wet-to-dry weight ratio (W/D) analysis, the left lung lobe was resected and its weight recorded. The tissue was then dried in a 60 °C oven for 72 h until a constant weight was achieved.

### Enzyme-linked immunosorbent assay

Blood samples were centrifuged at 3000 × g for 15 min to obtain serum. Cell culture supernatants were also collected for analysis. Serum IL-1β, TNF-α, and IL-6 levels were measured according to the manufacturer’s instructions. Absorbance at 450 nm was measured using a microplate reader (Epoch, Agilent).

### Cell culture maintenance and viability analysis

The mouse alveolar macrophage (AMs) cell line MH-S (Procell, CL-0597) was maintained in RPMI-1640 complete medium supplemented with 10% FBS, 0.05 mM β-mercaptoethanol, and 1% penicillin–streptomycin. The MLE-12 mouse alveolar epithelial cell line (iCell, SNL-414) was cultured in RPMI-1640 medium containing 10% FBS. Cells were incubated at 37 °C with 5% CO_2_ humidity.

A co-culture system was established using a 0.4 µm pore-sized Transwell insert to model the cellular interactions between MH-S and MLE-12 cells. MH-S cells were seeded in the lower chamber of a 6-well plate at a density of 1 × 10^5^ cells per well and treated with LPS (1 µg/mL) and YHJF (10%) for 24 h. The supernatant was then replaced with fresh medium before MLE-12 cells were placed in the upper chamber for a 12 h co-culture period. Finally, both supernatant and cells were harvested for subsequent analysis.

Cell viability was assessed using a Cell Counting Kit-8 (CCK-8; NCM Biotech, C6005). Following a 24-h stimulation with LPS at a concentration of 1 µg/mL and subsequent drug treatment, YHJF-containing serum at 5%, 10%, 15%, 20%, 25%, and 30% was added. CCK-8 solution was added per well followed by 1 h incubation at 37 °C. Absorbance at 450 nm was measured using a microplate reader (Epoch, Agilent).

### Detection of mitochondrial membrane potential, ROS, and ATP

Mitochondrial membrane potential (MMP) was assessed by JC-1 staining. Fresh lung tissue was minced and enzymatically digested at 37 °C for 30 min to prepare single-cell suspensions. Following 20 min incubation with JC-1 staining solution and three washes to remove unbound dye, samples were analyzed by flow cytometry using FITC and PE channels for JC-1 monomer and aggregate fluorescence detection, respectively. MH-S cells harvested 12 h post-modeling were stained with JC-1 for 20 min, washed three times with JC-1 buffer, and mitochondrial fluorescence intensity (red/green ratio) was quantified by confocal microscopy (STELLARIS, Leica).

For ATP detection, ATP lysis buffer was added to the samples (fresh lung tissue or MH-S cells). The lysates were centrifuged at 12,000 g at 4 °C for 10 min to obtain the clarified supernatant. The supernatant was then mixed with the ATP working solution in a 96-well plate. Chemiluminescence was detected using a microplate reader to quantify ATP levels.

Single-cell suspensions of lung tissue or MH-S cells were collected 12 h post-modelling were co-incubated with ROS staining solution in a 37 °C incubator for 2 h. Following washing to remove the unbound dye, fluorescence intensity was quantified by flow cytometry using the FITC channel.

### Transmission electron microscopy of MH-S cells and BALF-derived cells

For transmission electron microscopy(TEM), MH-S cells and BALF-derived cells were both collected by centrifugation at 500 g for 5 min, washed twice with PBS, and fixed with 2.5% glutaraldehyde in 0.1 M phosphate buffer (pH 7.4) at room temperature for 1 h. The samples were then dehydrated through a graded alcohol series (50% → 70% → 80% → 90% → 100%) and embedded in embedding resin (Epon 812). Thin sections were prepared and stained with 2.6% lead citrate. The morphology of mitochondria and the number of autophagosomes were observed using transmission electron microscope (TEM, JEOL, EM-1400, Tokyo, Japan).

### Immunofluorescence of MH-S and BALF-derived cells

MH-S cells and BALF-derived cells were both collected by centrifugation at 500 g for 5 min, washed twice with PBS, and spotted onto glass slides by cytocentrifugation (StatSpin Cytofuge 12, Thermo Scientific) at 400 rpm for 10 min. The cells were then blocked with serum and co-stained using a TSA kit (Servicebio, G1259-50T). Primary antibodies against PINK1 (1:1000), p-STING (1:1000), TOM20 (1:1000) was applied and incubated overnight at 4 °C. Following antigen retrieval, Parkin (1:1000), p-TBK1 (1:1000), and TFAM (1:800) were added, and the samples were incubated overnight. After washing with PBS, the samples were incubated with fluorescent secondary antibodies for 1 h. The samples were then washed, stained with 4',6-diamidino-2-phenylindole for nuclei visualisation, and mounted with antifade reagent. Co-localization was observed using confocal fluorescence microscopy (ECLIPSE Ti2-E, Nikon).

### Western blotting

Protein samples from lung tissue and primary cells were prepared using radioimmunoprecipitation assay (PRPA) lysis buffer containing phosphatase and protease inhibitors. After quantification and denaturation, proteins were separated by sodium dodecyl sulfate polyacrylamide gel electrophoresis (SDS-PAGE), transferred to a polyvinylidene fluoride membrane, and blocked using 5% bovine serum albumin (BSA) at room temperature for 2 h [31, 32]. PINK1 (1:2000), Parkin (1:1000), LC3-II (1:1000), p62 (1:1000), p-TBK1 (1:1000), TBK1 (1:1500), p-STING (1:1000), STING (1:1000), p-IRF3 (1:1000) were incubated overnight at 4 °C, followed by Tris-buffered saline with Tween 20 (TBST) washes (5 × 5 min). HRP-conjugated secondary antibody (Affinity, S0001) was incubated for 1 h, and the membrane was washed again with TBST (5 × 5 min). enhanced chemiluminescence reagent was used for visualisation, and band intensities were quantified using ImageJ (1.53, NIH).

### Total RNA isolation and quantitative real-time PCR (qRT-PCR)

Total RNA was extracted from tissues and cells using the SteadyPure Universal RNA Extraction Kit and reverse-transcribed to cDNA using the Evo M-MLV Reverse Transcription Premix Kit, initial denaturation at 95 °C for 30 s (1 cycle), followed by 40 cycles of denaturation at 95 °C for 5 s and extension at 60 °C for 30 s. mRNA expression levels were analysed using the 2^−ΔΔCT^ method with β-actin as the reference gene [18, 37]. The primer sequences used in this study are listed in Table S1.

### Pharmacological modulation of mitophagy in MH-S cells

Mdivi-1 (20 μM, mitophagy inhibitor) and urolithin A (UA; 10 μM, mitophagy activator) stock solutions in DMSO (500 mM for Mdivi-1 and 1000 mM for UA), stored at −20 °C, and diluted in complete medium before use (final DMSO ≤ 0.1%) [8, 53]. MH-S cells were divided into eight groups: Control (CON), CON + Mdivi-1, LPS, LPS + Mdivi-1, LPS + UA, LPS + YHJF, LPS + YHJF + Mdivi-1, and LPS + YHJF + UA. Cells were pretreated with Mdivi-1 or UA for 12 h, followed by LPS (1 μg/mL) and/or YHJF-containing (10%) for an additional 12 h. Mitochondrial function, Western blotting, qRT-PCR, and immunofluorescence were then performed.

### Apoptosis assay

Cell apoptosis was detected using the Annexin V-FITC/PI Apoptosis Detection Kit. Cells were harvested and washed twice with 4 °C ice-cold PBS. The cell density was adjusted to 1 × 10^6^ cells/mL to prepare single-cell suspensions. To each 100 μL aliquot of the cell suspension, 5 μL of FITC- Annexin V and 5 μL of propidium iodide were sequentially added. The mixture was then incubated in the dark for 5 min. Subsequently, 200 μL of binding buffer was added, and the sample was immediately analysed using a flow cytometer (CytoFLEX S, Beckman Coulter).

### Proteomics and bioinformatics enrichment analysis

Protein samples were prepared from mouse lung tissues(n = 6) and MH-S cells through ultrasonic processing ( CON group n = 6; LPS group and YHJF group n = 8). The collected protein samples were treated with triethylammonium bicarbonate and dispersed via ultrasonic vibration. Proteins were digested with trypsin. For detailed analysis, the peptides were subjected to liquid chromatography-tandem mass spectrometry ((LC–MS/MS). Peptide separation and high-resolution analysis of precursor ions and their fragments were conducted using the Vanquish UHPLC System (Thermo Scientific, Waltham, MA) in combination with the Q-Exactive HFX mass spectrometer (Thermo Fisher Scientific, Bremen, Germany).

Functional enrichment analysis was performed using GO and Reactome database to identify the most enriched biological processes (BP), cellular components (CC), molecular functions (MF), and key signalling pathways. Intersection targets were imported into the Metascape platform for GO and Reactome analysis and visualisation to explore the potential mechanisms of YHJF against SALI. Differentially expressed proteins (DEPs) from the proteomic analysis were identified based on a statistical significance threshold of *P* < 0.05 and a fold-change criterion of |*log*_*2*_*FC*|≥ 1. The Benjamini–Hochberg method controlled the False Discovery Rate (FDR), considering only DEPs with an FDR-adjusted *P* < 0.05 as significant for further analysis.

### CIBERSORT analysis of immune cell infiltration

Immune cell infiltration in lung tissues was analyzed using the CIBERSORT algorithm. Protein expression data were mapped to corresponding gene symbols and normalized to meet CIBERSORT input requirements [3, 48]. The analysis was performed with the standard LM22 signature matrix on the bioinformatics platform Sangerbox (http://www.sangerbox.com), and the proportion of immune cells in each sample was calculated. Results are presented as relative fractions of immune cell types.

### Molecular docking

Protein Data Bank (PDB) identifiers for receptor proteins. The receptor proteins were then modified using AutoDockTools (4.2.6,The Scripps Research Institute). The Grid programme’s Grid Option was used to process the receptor protein and convert them to pdbqt format. Molecular docking between receptor proteins and core blood-absorbed components of YHJF were performed with AutoDock Vina to score binding energies, with lower binding energies indicating stronger interactions.

### Data presentation and statistical analysis

All graphical representations were produced utilizing GraphPad Prism version 10.0 (San Diego, CA, USA). Statistical analyses were conducted using one-way ANOVA with Bonferroni correction. Statistical significance was defined as *P < 0.05 and **P < 0.01 when compared to the control, unless specified otherwise, and as #P < 0.05 and ##P < 0.01 when compared to the model. Survival curves were analyzed using the Kaplan–Meier method followed by log-rank tests with Bonferroni correction for multiple comparisons (α = 0.05).

## Results

### YHJF alleviates acute lung injury and inflammation in CLP-induced SALI mice

**Fig. 1:**
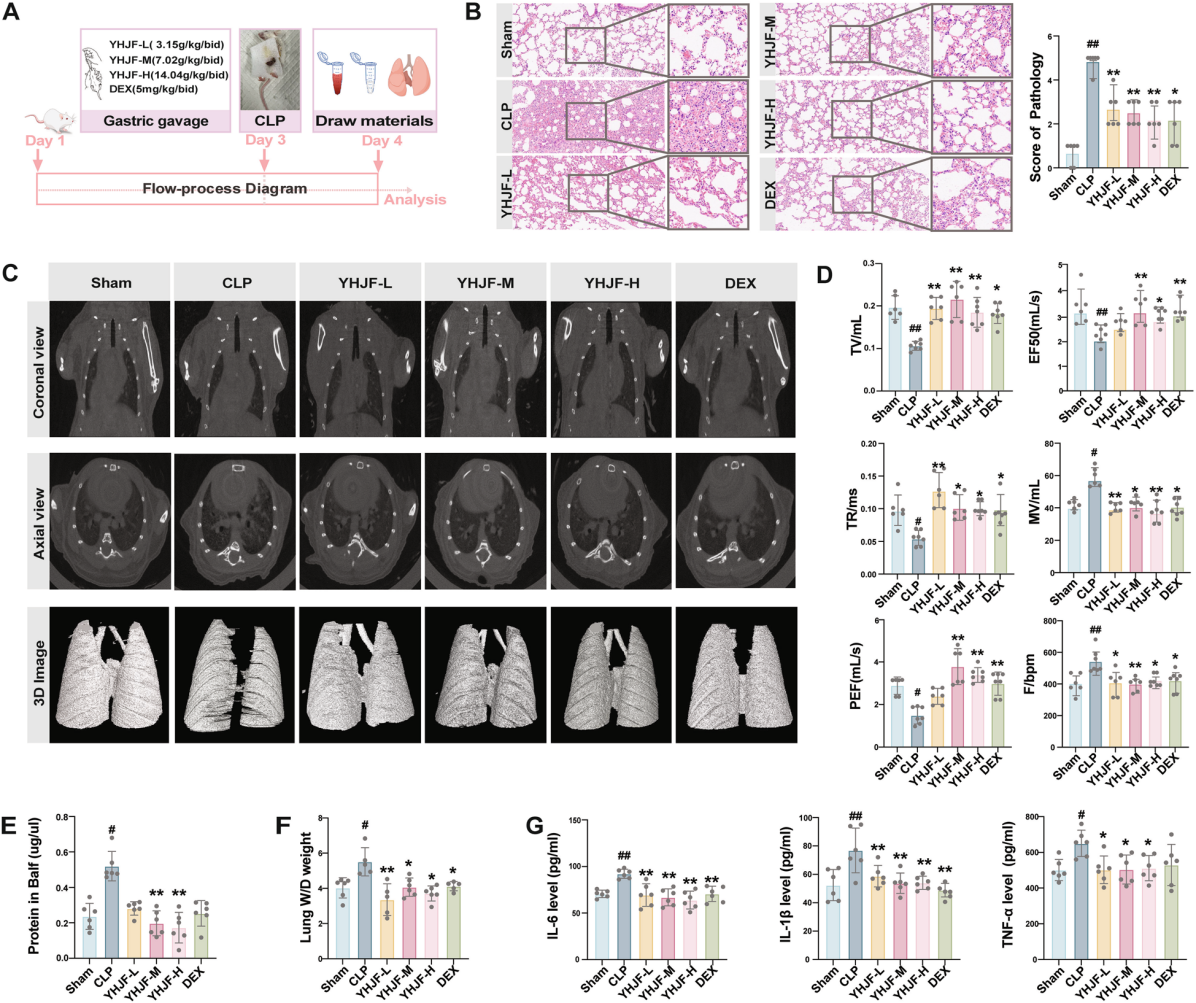
YHJF alleviates acute lung injury in CLP induced septic mice. **A** Schematic diagram of the animal experimental procedure; **B** HE staining shows lung tissue pathological damage and injury scores; **C** Micro-CT imaging of lung tissue; **D** Lung function parameters (respiratory frequency, tidal volume, lung compliance) measured by a ventilator; **E** Wet/dry weight ratio (W/D) of lung tissue; **F** Total protein content in bronchoalveolar lavage fluid; **G** mRNA levels of inflammatory cytokines (IL-1β, IL-6, and TNF-α) in mouse serum detected by qRT-PCR. Data are presented as the mean ± SD (n = 6). *P < 0.05, **P < 0.01 vs. CLP group; #*P* < 0.05, ##*P* < 0.01 vs. Sham group

Mice were pretreated with YHJF or DEX for 3 days before CLP surgery (Fig. 1A).Survival analysis demonstrated a significant benefit of YHJF over untreated CLP control (SFig. 1). Histopathological evaluation of lung tissues revealed severe alveolar damage in CLP mice, marked by alveolar septal thickening, inflammatory cell infiltration, and extensive tissue injury, whereas these pathological changes were markedly attenuated by YHJF treatment (*P* < 0.01 vs. CLP group; Fig. 1B). Consistently, micro-CT imaging confirmed substantial pulmonary injury in CLP mice, while YHJF treatment showed a clear protective effect against lung damage (Fig. 1C). Functional assessment demonstrated that YHJF significantly improved respiratory rate, tidal volume, and lung compliance in septic mice (*P* < 0.05 to *P* < 0.01 vs. CLP group; Fig. 1D). In addition, reduced BALF protein content and lung wet/dry ratios indicated alleviated pulmonary edema in YHJF-treated mice (*P* < 0.05 to *P* < 0.01 vs. CLP group; Fig. 1E, F).

Serum cytokine profiling via ELISA showed elevated IL-6, IL-1β, and TNF-α levels in the CLP group (*P* < 0.05 to *P* < 0.01 vs. Sham group), which were suppressed by both YHJF and DEX (*P* < 0.05 to *P* < 0.01 vs. CLP group; Fig. 1G). Collectively, these data suggest that CLP -induced SALI mice develop severe pulmonary damage, whereas YHJF significantly alleviates mice lung injury, pulmonary edema, and systemic inflammatory responses.

### YHJF modulates mitochondrial homeostasis and macrophage function in CLP induced SALI mice by proteomic analysis

**Fig. 2:**
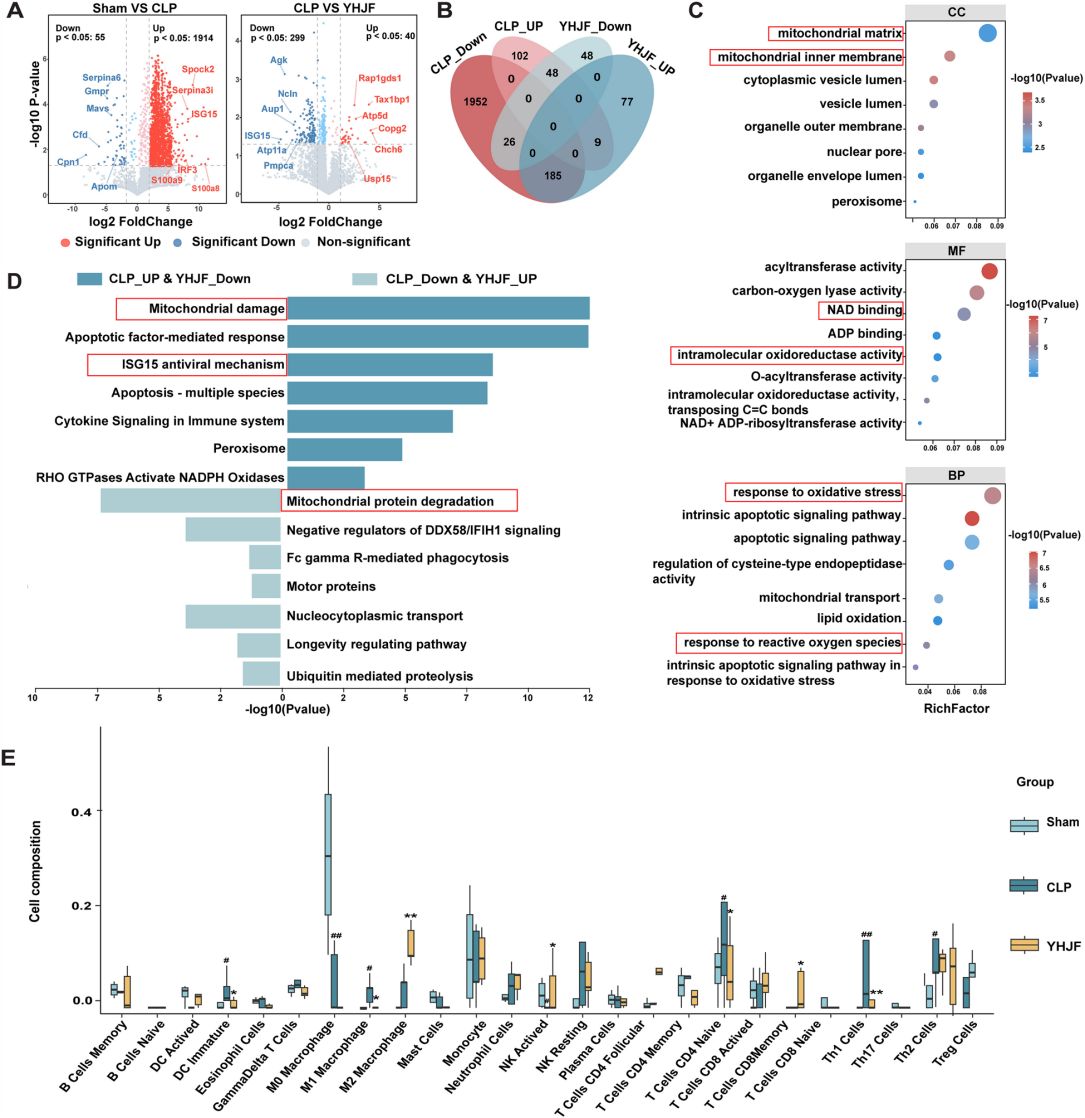
YHJF improves mitochondrial dysfunction in SALI mice. **A** Volcano plots display DEPs in lung tissues comparing the Sham group with the CLP group, and the CLP group with the YHJF group (n = 6); **B** The Venn diagram shows the overlap of up- and down-regulated DEPs between the CLP and Sham groups, and the YHJF and CLP groups; **C** GO enrichment analysis, including cellular components (CC), molecular functions (MF), and biological processes (BP); **D** Reactome pathway enrichment analysis; **E** Immune infiltration analysis based on lung proteomics. **P < 0.01 vs. CLP group; #*P* < 0.05, ##*P* < 0.01 vs. Sham group

To explore the underlying mechanism of YHJF therapeutic efficacy, proteomic analysis was first conducted on murine lung tissues. Mass spectrometrybased proteomic analysis identified a total of 2908 proteins in septic murine lung tissues. To focus on the most significantly altered proteins, we applied strict criteria and identified 268 core DEPs for further analysis (|*log*_*2*_*FC*|≥ 1, *FDR* < 0.05; Fig. 2A). As depicted in the Venn diagram (Fig. 2B), among these DEPs, 48 proteins were downregulated in the CLP group but upregulated following YHJF treatment, whereas 185 proteins demonstrated the inverse pattern, suggesting potentially reversible and treatment-responsive alterations. Furthermore, 26 proteins were consistently downregulated in both the CLP and YHJF groups, while 9 proteins remained upregulated under both conditions.

Gene Ontology (GO) enrichment analysis of the 268 core DEPs indicated that these proteins were mainly associated with mitochondrial matrix, mitochondrial inner membrane, NAD binding and intramolecular oxidoreductase activity, response to oxidative stress and response to reactive oxygen species (Fig. 2C). Reactome pathway analysis further revealed that proteins downregulated in CLP but restored by YHJF were enriched in pathways related to mitochondrial protein degradation. Conversely, proteins upregulated by CLP but suppressed by YHJF were associated with mitochondrial damage and ISG15 antiviral pathways (Fig. 2D). These data suggest that YHJF may exert protective effects in septic lung injury by modulating mitochondrial homeostasis and oxidative stress-related processes.

The immune cell infiltration analysis revealed that macrophages were the predominant cell type altered in lung tissues after CLP-induced injury (Fig. 2E). Specifically, the CLP group showed a significant increase in pro-inflammatory macrophages (M1 macrophage) compared to the Sham group (*P* < 0.05). Notably, this elevation was markedly reversed by YHJF treatment (*P* < 0.05 vs. CLP group). These results pinpoint macrophages as a primary cellular target through which YHJF may mitigate immune dysregulation in sepsis-associated acute lung injury.

### YHJF mitigates mitochondrial dysfunction and modulates macrophage activity

**Fig. 3:**
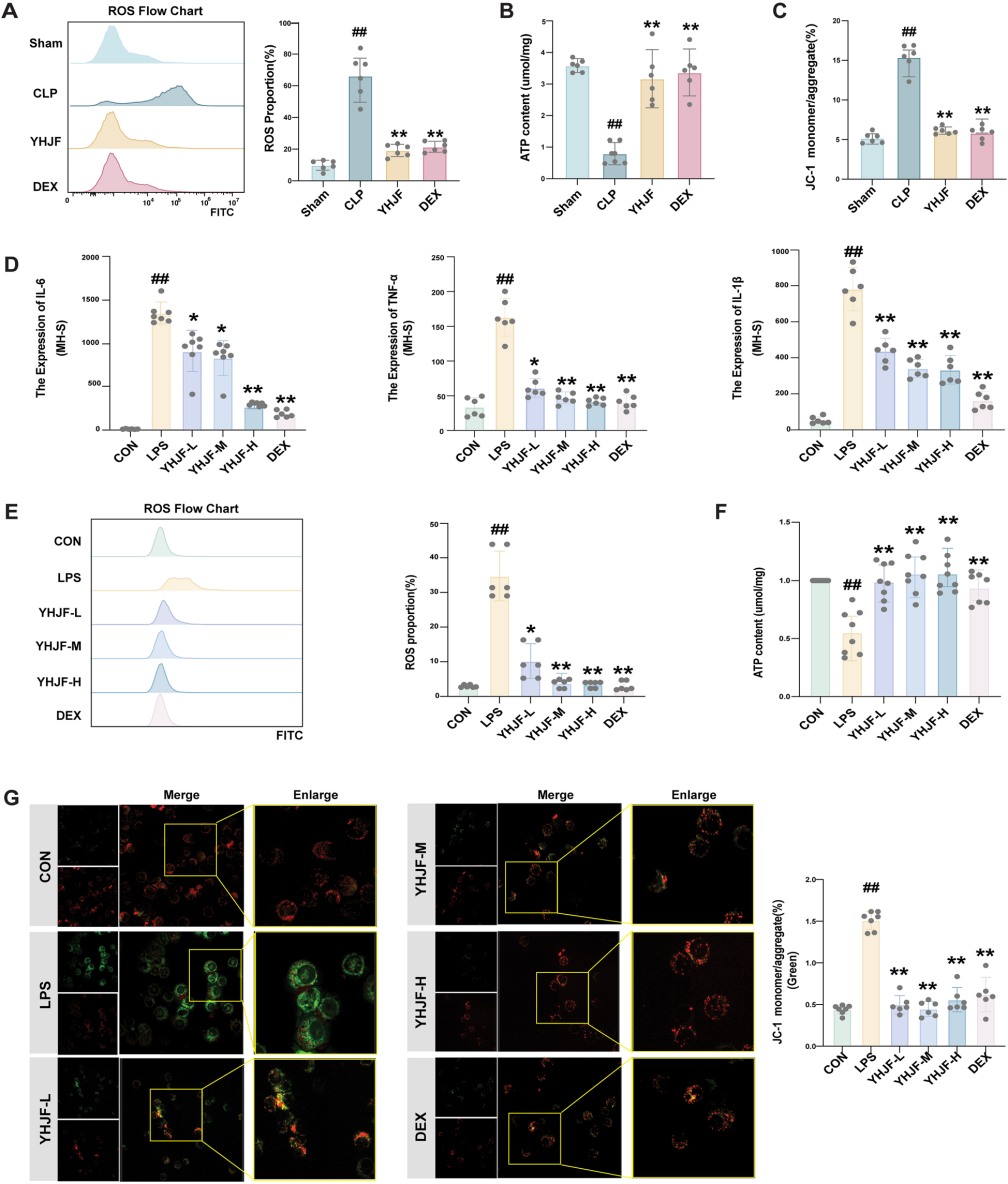
YHJF improves mitochondrial dysfunction in alveolar macrophages. **A** ROS expression level in lung tissue (n = 6); **B** ATP expression level in lung tissue (n = 6); **C** Detection of JC-1 in lung tissue. (n = 6). Data are presented as the mean ± SD.*P < 0.05, **P < 0.01 vs. CLP group; #*P* < 0.05, ##*P* < 0.01 vs. Sham group. **D** ELISA detects levels of IL-1β, IL-6, TNF-α in the supernatant of MH-S cells (n = 6); **E** Flow cytometry detects ROS fluorescence intensity in MH-S cells (n = 6); **F** ATP level in MH-S cells (n = 6); **G** JC-1 staining shows mitochondrial membrane potential in MH-S cells (green monomers, red aggregates) (n = 6). Data are presented as the mean ± SD. *P < 0.05, **P < 0.01 vs. LPS group; #*P* < 0.05, ##*P* < 0.01 vs. CON group

To validate our proteomic findings, we examined mitochondrial function in lung tissues. We found that CLP mice exhibited significantly elevated ROS fluorescence intensity (*P* < 0.01 vs. Sham group) and reduced ATP levels, both of which were reversed by YHJF treatment (*P* < 0.01 vs. CLP group; Fig. 3A, B). Furthermore, YHJF increased mitochondrial membrane potential, as demonstrated by an elevated JC-1 aggregate/monomer (red/green) ratio compared to the CLP group (*P* < 0.01 vs. CLP group; Fig. 3C).

To further validate these findings in vitro, we established an LPS induced MH-S cell model to simulate the macrophage damage. CCK-8 assays were performed to determine the optimal LPS concentration (1 μg/mL) and appropriate YHJF-containing serum concentrations for subsequent experiments (SFig. 2). YHJF significantly inhibited LPS stimulated secretion of IL-1β, TNF-α, and IL-6 (*P* < 0.05 to *P* < 0.01 vs. LPS group; Fig. 3D). Flow cytometry and biochemical assays demonstrated LPS stimulated mitochondrial dysfunction in MH-S cell, characterized by elevated ROS levels and reduced ATP production, both of which were significantly improved by YHJF treatment (*P* < 0.01 vs. LPS group; Fig. 3E, F). JC-1 staining further confirmed the restoration of mitochondrial membrane potential in YHJF-treated cells, as evidenced by increased JC-1 monomer ratios (*P* < 0.01 vs. LPS group; Fig. 3G). These results demonstrate that YHJF effectively alleviate mitochondrial dysfunction and inflammatory activation in AMs.

### *YHJF activates mitophagy to mitigate mitochondrial dysfunction both *in vitro* and *in vivo

To elucidate the molecular basis underlying the protective effects of YHJF on macrophage mitochondrial function, we performed proteomic analysis of MH-S cells. Principal component analysis (PCA) revealed distinct clustering among the CON, LPS and YHJF intervention groups (SFig. 3A). Comparative proteomic analysis revealed 398 DEPs between the LPS and CON groups (|*log*_*2*_* FC*|≥ 1, *FDR* < 0.05), whereas YHJF intervention significantly modulated 248 DEPs (SFig. 3B). Venn diagram analysis further identified 128 overlapping DEPs that were considered potential mediators of the protective effects of YHJF against LPS-induced macrophage injury (SFig. 3C).

Functional enrichment analyses were then performed to characterize the biological significance of these overlapping DEPs. Reactome pathway analysis revealed significant enrichment of these DEPs, including cytokine signaling in the immune system, interferon signalling, and mitophagy(animal) (SFig. 3D). Gene Ontology (GO) enrichment analysis further classified the DEPs into various categories: Biological Processes, including innate immune response, response to type II interferon, and regulation of ATP-dependent activity; Molecular Functions, specifically antioxidant activity; and Cellular Components, notably the mitochondrial outer membrane(SFig. 3E–G).

**Fig. 4:**
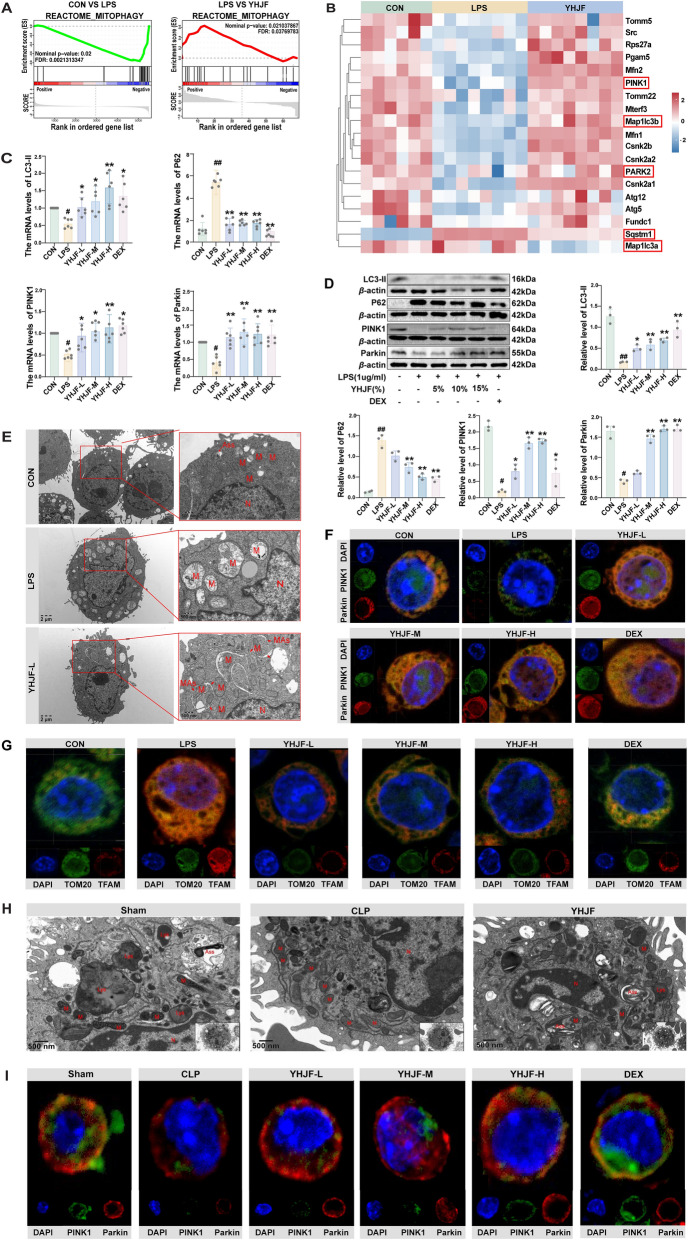
YHJF alleviate mitochondrial damage in alveolar macrophages by activating mitophagy. **A** GSEA analysis of MH-S cell proteome: mitochondrial pathway (CON group n = 6; LPS group and YHJF group n = 8); **B** Heatmap shows proteins related to the mitophagy pathway; **C** qRT-PCR detects mRNA levels of p62, LC3-II, PINK1, and Parkin in MH-S cells (n = 6); **D** Western blot detects protein expression levels of p62, LC3-II, PINK1, and Parkin in MH-S cells (n = 3); **E** TEM shows mitochondrial morphology and status in MH-S cells (n = 6); **F** PINK1 and Parkin immuno-co-localization confirms mitophagy localization in MH-S cells (n = 6); **G** TFAM and TOM20 immunofluorescence confirm mtDNA expression in MH-S cells (n = 6). **H** TEM revealed mitochondrial morphology and status in BALF-derived cells (n = 6); **I** PINK1 and Parkin immuno-co-localization confirms mitophagy localization in BALF-derived cells (n = 6). Data are presented as the mean ± SD. *P < 0.05, **P < 0.01 vs. LPS group; #*P* < 0.05, ##*P* < 0.01 vs. CON group

Furthermore, Gene set enrichment analysis (GSEA) in MH-S demonstrated significant enrichment of mitophagy related pathways following YHJF treatment compared to LPS group (*P* < 0.05; Fig. 4A). The heat map further revealed distinct expression patterns of mitophagic regulators across the experimental groups (Fig. 4B). The hypothesis that mitophagy plays a critical role in macrophages and may underlie the protective effects of YHJF was then tested experimentally. Validation by qRT-PCR and western blot analysis showed that LPS stimulation significantly downregulated the expression of PINK1, Parkin, and LC3-II, while promoting p62 accumulation, compared with the control group. Both YHJF and DEX reversed these molecular changes (*P* < 0.05 to *P* < 0.01 vs. LPS group; Fig. 4C, D). TEM analysis of LPS challenged MH-S cells demonstrated that YHJF treatment effectively attenuated mitochondrial ultrastructural abnormalities, including swelling and cristae disorganisation, while significantly increasing autophagosome-engulfed mitochondria (Fig. 4E). Quantitative analysis using confocal microscopy revealed that LPS treatment significantly impaired the colocalisation of PINK1 and Parkin in cells, whereas this alteration was markedly ameliorated by YHJF intervention (Fig. 4F). Moreover, LPS stimulation significantly increased cytosolic mtDNA release, whereas YHJF treatment effectively reduced mtDNA-positive cytoplasmic signals in MH-S cells (Fig. 4G). Together, these results indicate that YHJF alleviates mitochondrial damage while promoting mitophagy in LPS-stimulated macrophages.

Transmission electron microscopy (TEM) of BALF-derived cells (predominantly alveolar macrophages) revealed extensive mitochondrial vacuolization and marked ultrastructural damage in the CLP group compared to the Sham group. In contrast, YHJF treatment significantly reduced mitochondrial swelling and increased autophagosome formation, indicating enhanced mitophagy and autophagolysosome maturation (Fig. 4H). Furthermore, immunofluorescence co-localization analysis demonstrated a significant increase in PINK1/Parkin co-localization in BALF cells from the YHJF group compared to the CLP group (Fig. 4I). Together, these results demonstrate that YHJF attenuates mitochondrial injury and restores impaired mitophagic flux in alveolar macrophages within the SALI mouse model.

### YHJF suppresses mtDNA release and STING pathway activation

**Fig. 5:**
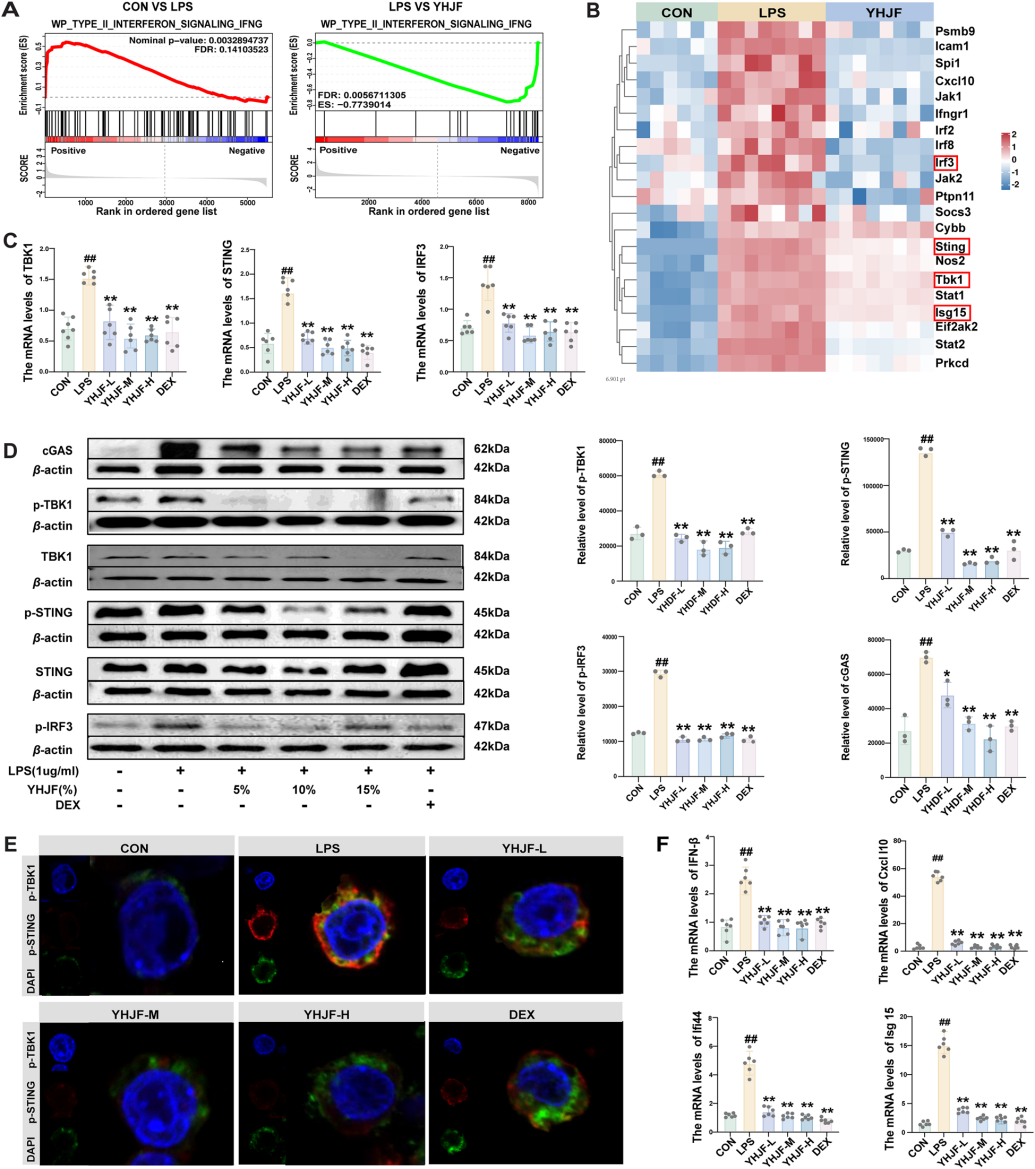
YHJF inhibits activation of the mtDNA–STING inflammatory axis in LPS-stimulated MH-S macrophages. **A** MH-S cell proteome GSEA analysis—type I interferon signaling pathway (CON group n = 6; LPS group and YHJF group n = 8); **B** Heatmap of proteomics analysis; **C** qRT-PCR analysis of TBK1, STING, and IRF3 mRNA expression in MH-S cells (n = 6); **D** Western blot analysis of cGAS、p-TBK1, p-STING and p-IRF3 expression in MH-S cells (n = 3); **E** Co-localisation of p-TBK1 and p-STING fluorescence signals (n = 6); **F** qRT-PCR analysis of IFN-β, Ifi44, Cxcl10, and Isg15 in MH-S cells (n = 6). Data are presented as the mean ± SD. *P < 0.05, **P < 0.01 vs. LPS group; #*P* < 0.05, ##*P* < 0.01vs. CON group

To assess whether YHJF-induced mitophagy activation reduces mtDNA leakage, suppressing the mtDNA-STING inflammatory pathway and providing protection. We next performed GSEA based on the proteomic profiles of MH-S cells. The analysis showed significant enrichment of the type I interferon signaling pathway in LPS-treated cells, whereas this enrichment was markedly attenuated after YHJF treatment (*P* < 0.01; Fig. 5A). A heatmap further visualized the expression trends of pathway-related targets across groups (Fig. 5B).

Consistent with the enrichment results, qRT-PCR analysis demonstrated YHJF significantly downregulated the expression of STING, TBK1, and IRF3 proteins compared to the LPS group (*P* < 0.01; Fig. 5C). At the protein level, YHJF decreased the expression of cGAS, and reduced the phosphorylation levels of STING, TBK1, and IRF3 (*P* < 0.05 to *P* < 0.01 vs. LPS group; Fig. 5D), without altering their total protein expression.. Confocal microscopy showed enhanced spatial colocalisation of phosphorylated STING (p-STING) and TBK1 (p-TBK1) in LPS-treated MH-S cells (Fig. 5E). To further validate this mechanism, qRT-PCR analysis of downstream pathway related factors confirmed that YHJF markedly inhibited the up-regulation of IFN-β, Ifi44, Cxcl10, and Isg15 in MH-S cells (*P* < 0.01 vs. LPS group; Fig. 5F). These experimental results collectively demonstrate that YHJF confers protection by activating mitophagy to eliminate damaged mitochondria and by downregulating the mtDNA-driven activation of the STING inflammatory pathway.

### Mitophagy mediates YHJF-induced mitochondrial protection in LPS-stimulated macrophages

**Fig. 6:**
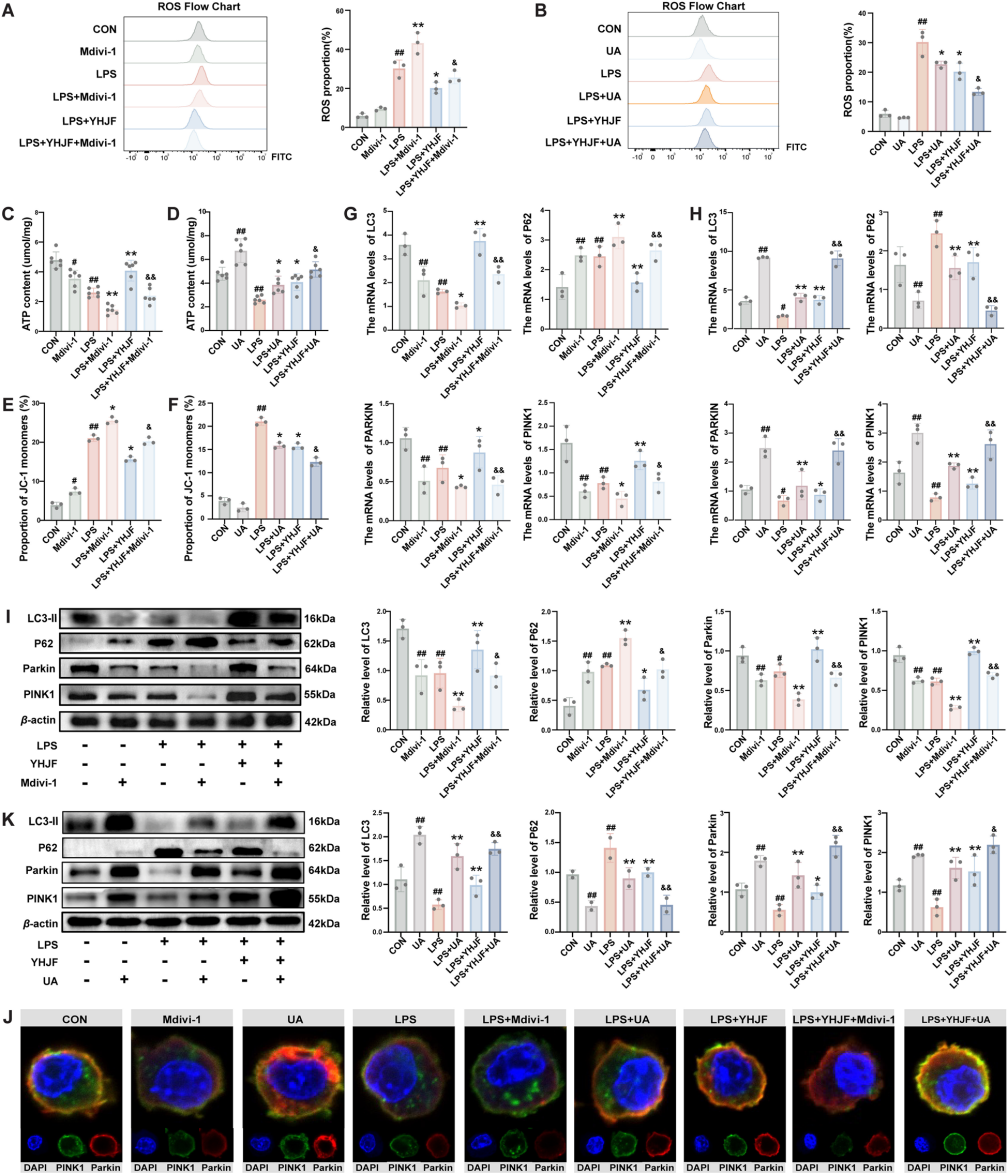
Pharmacological validation of mitophagy involvement in YHJF-mediated mitochondrial protection. **A**, **B** Flow cytometric analysis and quantification of intracellular ROS levels (n = 3); **C**, **D** ATP content and quantitative analysis (n = 3); **E**, **F** JC-1 monomer ratio for assessment of mitochondrial membrane potential (n = 3); **G**, **H** qRT-PCR analysis and quantification of mitophagy-related mRNA levels for PINK1, Parkin, LC3B, and p62 (n = 3);; **I**, **J** Western blot analysis and quantification of mitophagy-related proteins levels for PINK1, Parkin, LC3-II, and p62(n = 3); **K** Representative immunofluorescence images showing colocalization of PINK1 and Parkin in MH-S cells (n = 3). Data are presented as mean ± SD. #*P* < 0.05 vs. CON group; *P < 0.05 vs. LPS group; &*P* < 0.05 vs. LPS + YHJF group

To investigate the role of mitophagy in the mitochondrial protection conferred by YHJF, we modulated mitophagy pharmacologically in LPS‑stimulated MH‑S cells using the inhibitor Mdivi‑1 and the activator UA. Relative to the LPS group, YHJF co‑treatment significantly lowered intracellular ROS, increased ATP production, and reduced the JC‑1 monomer ratio. These improvements were partly reversed by Mdivi‑1, which elevated ROS, decreased ATP, and raised the JC‑1 monomer ratio compared to the LPS + YHJF group (*P* < 0.05 to *P* < 0.01). In contrast, UA further enhanced the protective effects of YHJF, yielding even lower ROS and JC‑1 monomer levels and higher ATP than the LPS + YHJF group (all *P* < 0.05; Fig. 6A–F).

Consistent with these functional changes, YHJF upregulated mRNA expression of PINK1, Parkin, and LC3II and downregulated p62, as shown by qRT‑PCR. Mdivi‑1 blunted these YHJF‑induced transcriptional changes, whereas UA amplified them (all *P* < 0.01 vs. LPS + YHJF group; Fig. 6G, H). Western blot analysis mirrored these findings at the protein level: YHJF increased PINK1, Parkin, and LC3II proteins and decreased p62 accumulation. Again, Mdivi‑1 suppressed these alterations, while UA enhanced them (*P* < 0.05 to *P* < 0.01 vs. LPS + YHJF group; Fig. 6I, J). Immunofluorescence co‑localization of PINK1 and Parkin further confirmed that the YHJF‑induced increase in co‑localization was reduced by Mdivi‑1 and augmented by UA (Fig. 6K).

Collectively, the opposing actions of Mdivi‑1 and UA on mitochondrial parameters, mitophagy markers, and PINK1/Parkin co‑localization establish mitophagy as a critical mechanism underlying the mitochondrial protection exerted by YHJF in LPS‑stimulated macrophages.

### YHJF suppresses the mtDNA-STING inflammatory axis in a mitophagy-dependent mechanisms

**Fig. 7:**
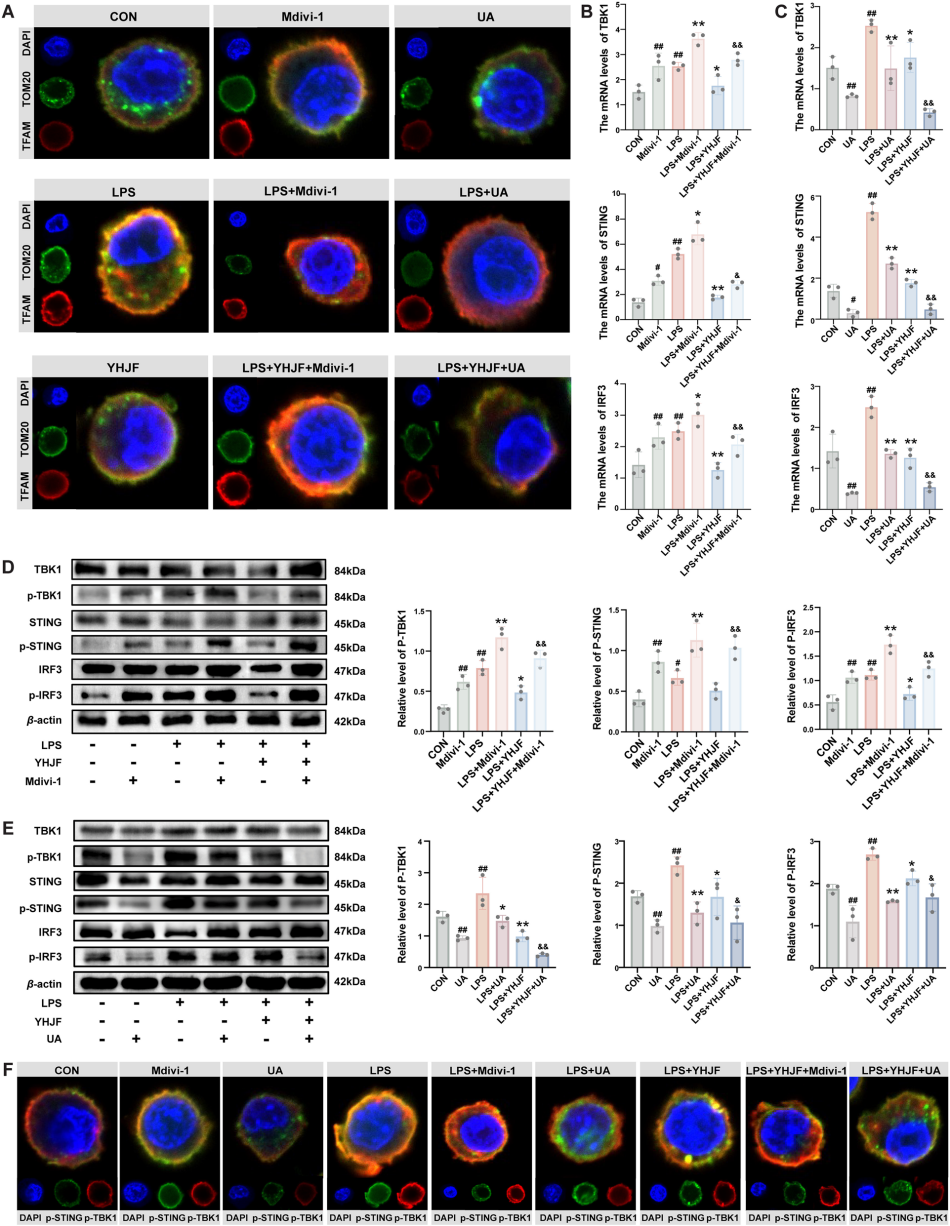
YHJF suppresses mtDNA-STING pathway activation in a mitophagy-linked manner in LPS-stimulated MH-S cells. **A** Representative immunofluorescence images showing colocalization of TFAM and TOM20 in MH-S cells(n = 3). **B**, **C** qRT-PCR analysis of TBK1, STING and IRF3 mRNA expression(n = 3). **D**, **E** Western blot analysis and quantification of total and phosphorylated TBK1, STING and IRF3(n = 3). **F** Representative immunofluorescence images showing colocalization of p-TBK1 and p-STING in MH-S cells(n = 3). Data are presented as mean ± SD. #*P* < 0.05 vs. CON group; *P < 0.05vs. LPS group; &*P* < 0.05 vs. LPS + YHJF group

To determine whether YHJF suppresses the mtDNA–STING inflammatory axis via mitophagy activation, we performed bidirectional pharmacological modulation using the mitophagy inhibitor Mdivi‑1 and activator UA, and assessed mtDNA retention and STING signaling. Immunofluorescence analysis of TOMM20 and TFAM colocalization showed that YHJF restored the LPS‑disrupted colocalization. This effect was weakened by Mdivi‑1 and further enhanced by UA (Fig. 7A). qRT‑PCR demonstrated that YHJF suppressed LPS‑induced upregulation of Sting, Tbk1, and Irf3 mRNA (*P* < 0.05 vs. LPS group). Co‑treatment with Mdivi‑1 partially reversed this suppression, whereas UA alone reduced their expression and UA co‑treatment further potentiated the YHJF‑mediated inhibition (*P* < 0.05 to *P* < 0.01 vs. LPS + YHJF group; Fig. 7B, C). Western blot analysis showed that total STING, TBK1, and IRF3 protein levels remained unchanged across groups, while LPS robustly increased their phosphorylation. YHJF significantly reduced p‑STING, p‑TBK1, and p‑IRF3 levels (*P* < 0.05 vs. LPS group); this reduction was attenuated by Mdivi‑1 and further strengthened by UA (*P* < 0.05 to *P* < 0.01 vs. LPS + YHJF group; Fig. 7D, E). Similarly, immunofluorescence revealed that YHJF decreased LPS‑enhanced colocalization of p‑STING and p‑TBK1, an effect reversed by Mdivi‑1 and further diminished by UA (Fig. 7F).

In summary, pharmacological inhibition of mitophagy by Mdivi‑1 weakened the inhibitory effect of YHJF on the mtDNA–STING pathway, whereas mitophagy activation by UA enhanced it, confirming that YHJF acts, at least in part, through promoting mitophagy.

### YHJF alleviates alveolar epithelial cell damage by modulating macrophage activity in a co-culture system

**Fig. 8:**
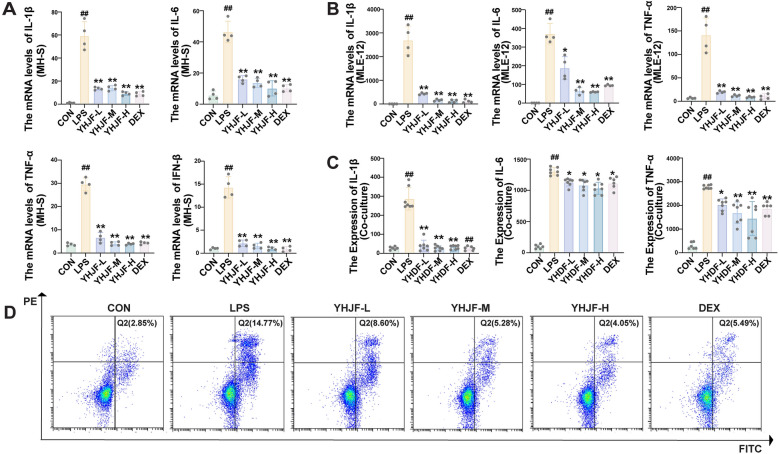
YHJF reduces alveolar epithelial cell damage by adjusting macrophage-driven inflammation in a co-culture system. **A** qRT-PCR analysis for expression of IL-1β, IL-6, TNF-α, and IFN-β in MH-S (n = 6). **B** qRT-PCR analysis for expression of IL-1β, IL-6, TNF-α in MH-S. **C** Levels of IL-1β, IL-6 and TNF-α in co-culture supernatants (n = 6). **D** Flow cytometric analysis and quantification of apoptosis in MLE-12 cells following co-culture with MH-S (n = 6). Data are presented as the mean ± SD. *P < 0.05, **P < 0.01 vs. LPS group; #*P* < 0.05, ##*P* < 0.01 vs. CON group

To elucidate the role of MH-S macrophages in sepsis-induced lung injury and the regulatory effects of YHJF, we established a co-culture system comprising MH-S macrophages and MLE-12 alveolar epithelial cells. After 12 h of co-culture, qRT-PCR analysis revealed a significant upregulation of IL-6, IL-1β, and TNF-α mRNA levels in MH-S cells following LPS stimulation. A similar increase in the expression of these cytokines was also observed in MLE-12 cells. Notably, treatment with YHJF significantly attenuated the LPS-induced upregulation of IL-6, IL-1β, and TNF-α in both cell types. Additionally, YHJF suppressed LPS-triggered IFN-β gene expression (*P* < 0.05 to *P* < 0.01 vs. LPS group; Fig. 8A, B). These findings were further corroborated by ELISA analysis of the co-culture supernatants, which demonstrated that YHJF effectively inhibited the secretion of IL-6, IL-1β, and TNF-α (*P* < 0.05 to P < 0.01 vs. LPS group; Fig. 8C). The inhibitory effect of YHJF on cytokine expression was dose-dependent.

Flow cytometry analysis further demonstrated that LPS stimulation significantly increased apoptosis in MLE-12 cells within the macrophage co-culture system, whereas YHJF treatment effectively suppressed this increase (Fig. 8D). These results suggest that YHJF mitigates epithelial cell damage by modulating macrophage-mediated inflammatory cascades and attenuating apoptosis.

### Molecular docking prediction of YHJF components regulating macrophage mitophagy in SALI

Comprehensive mass spectrometry analysis of the YHJF formulation was performed using dual-polarity (positive/negative) ion chromatograms to systematically characterise its chemical profile (SFig. 4). This analysis led to the annotation and identification of 101 chemical constituents (Table S2), including 41 blood-absorbed components (Table S3).

**Fig. 9:**
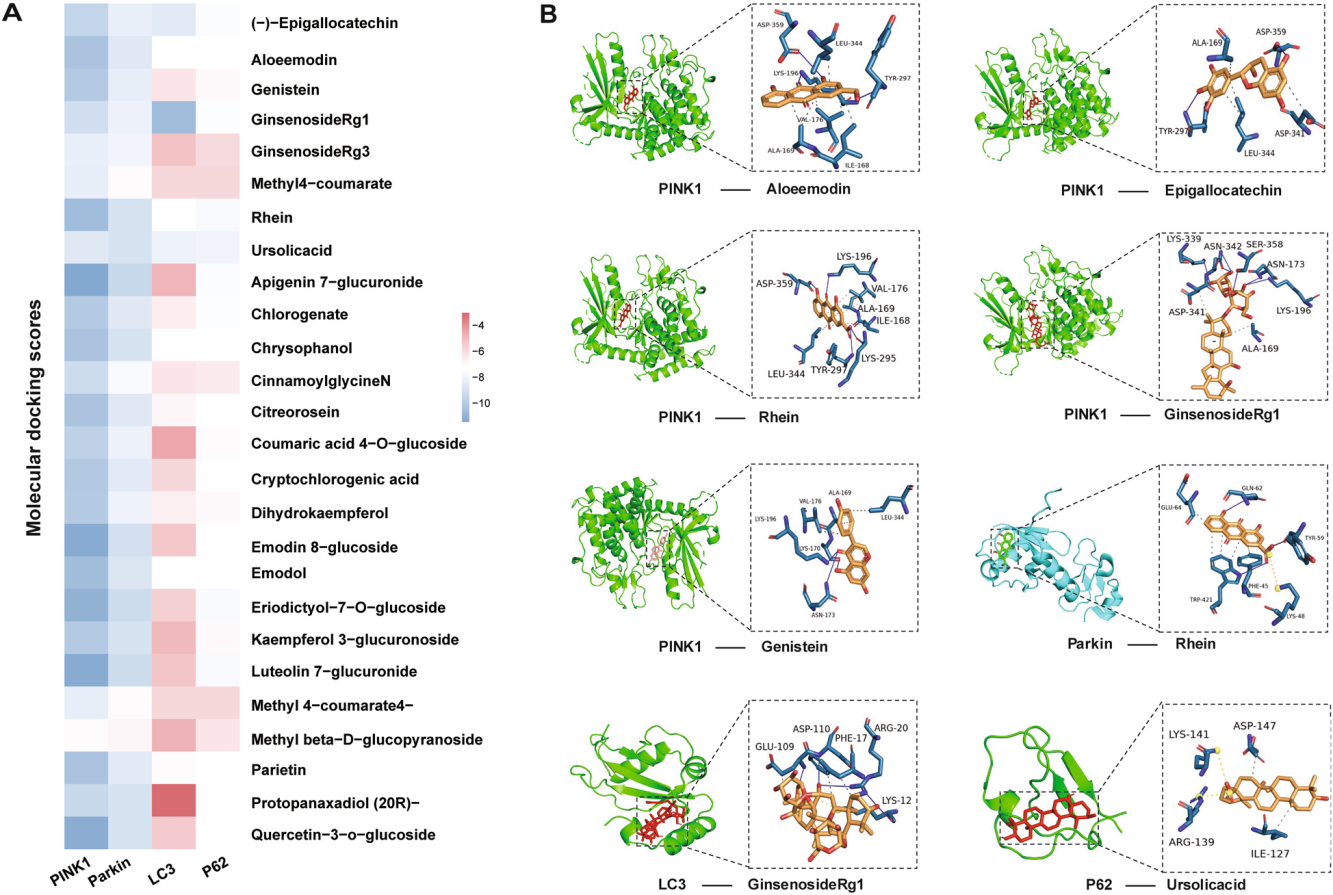
Molecular docking validation of the core mechanism of yhjf in alleviating sepsis-induced acute lung injury. **A** Heatmap of binding scores between core YHJF components and mitophagy targets (PINK1;Parkiin; LC3;P62). **B** Representative molecular docking results of the top-ranked stable ligand target pairs for each key target

To explore potential mechanisms, molecular docking simulations were conducted to evaluate the binding interactions between systemically absorbed YHJF components and key pharmacological targets. A heatmap visually highlights 26 constituents showing favorable binding affinities with major therapeutic targets (Fig. 9A, B), and quantitative docking energetics are summarized in Table S4, with lower binding energies indicating stronger molecular interactions; representative binding modes are displayed in Fig. 9B. Among the examined targets, PINK1 exhibited notably strong predicted binding affinity with several blood‑absorbed compounds. Additionally, aloe‑emodin, rhein, and genistein demonstrated favorable docking interactions with multiple mitophagy‑related proteins. These results indicate that YHJF contains active components with potential modulatory effects on mitophagy.

## Discussion

Sepsis is characterised by a dysregulated host response to infection and remains a major cause of mortality in critically ill patients [13]. SALI is among the most frequent and severe complications of sepsis and is characterised by pulmonary inflammation, oedema, and progressive impairment of respiratory function [41]. Increasing evidence indicates that autophagy, particularly mitophagy, is an important adaptive mechanism in sepsis because it removes damaged mitochondria, limits ROS accumulation, and prevents the cytosolic release of mtDNA, a potent damage-associated molecular pattern that amplifies innate immune activation [4]. In SALI, defective mitophagy may initiate a vicious cycle of mitochondrial injury, oxidative stress, and persistent inflammation, ultimately aggravating pulmonary dysfunction [43]. Therefore, restoration of effective mitophagy may represent a promising therapeutic strategy for sepsis-induced lung injury.

Traditional Chinese medicine has shown increasing therapeutic promise in sepsis because of its multi-component and multi-target characteristics [49, 54]. In TCM theory, sepsis is characterized by deficiency, toxin, and stasis, and treatment is therefore directed toward reinforcing vital qi, promoting blood circulation, and eliminating toxins [10]. Based on this framework, Professor Li Jun, under the guidance of National Master of Traditional Chinese Medicine Chen Shaohong, advanced the clinical conceptualization of sepsis as a syndrome involving vital qi deficiency, toxin damage, and blood stasis, and proposed the therapeutic principle of “Yiqi, Huoxue, and Jiedu”. YHJF (Patent No.: ZL202111547052.1) was developed on this basis through modification of the classical Renshen Dahuang Decoction from Bian Zheng Lu, using Panax ginseng, Panax notoginseng, and Rheum palmatum in accordance with the principle of tonifying qi, activating blood circulation, and removing toxins. YHJF has been applied as an adjunctive therapy in sepsis for over two decades, and our previous studies showed that it improved clinical outcomes, regulated bile acid metabolism, and alleviated NKT-cell exhaustion in sepsis [5, 6, 16, 25]. These observations provided the rationale for the present study.

In this study, we found that YHJF markedly protected against SALI in vivo. YHJF significantly improved 7-day survival in CLP-induced septic mice and alleviated key manifestations of lung injury, including inflammatory infiltration, alveolar septal thickening, pulmonary oedema, and impaired respiratory function. Importantly, these protective effects were supported not only by conventional histological and biochemical indices, but also by micro-CT and pulmonary function assessment, indicating improvement in both lung structure and function. Dexamethasone is frequently used as a standard anti-inflammatory comparator in studies of pulmonary injury [52], and our findings further support the protective effect of YHJF in SALI. However, the benefit of YHJF may extend beyond non-specific anti-inflammatory suppression, suggesting a broader multi-target therapeutic profile.

A central finding of this study is that macrophage mitophagy appears to be a key target of YHJF. Proteomic analyses indicated significant enrichment of mitophagy-related pathways in lung tissue and macrophages after YHJF treatment. Consistent with this, both the CLP model and LPS-stimulated MH-S macrophages showed that YHJF reduced ROS accumulation, restored ATP production, preserved mitochondrial membrane potential, and upregulated PINK1, Parkin, and LC3B. In vivo validation using BALF-derived cells from CLP mice further revealed that YHJF treatment markedly increased autophagosome formation and enhanced the co-localization ratio of PINK1 and Parkin. Collectively, these results support the interpretation that YHJF alleviates SALI, at least in part, by augmenting mitophagy and protecting alveolar macrophages against mitochondrial damage.

An important strength of this study is the bidirectional pharmacological modulation of mitophagy using Mdivi-1 and urolithin A (UA). Mdivi-1, an inhibitor of mitochondrial fission and mitophagy, substantially attenuated the beneficial effects of YHJF on mitochondrial function, whereas UA, a recognised mitophagy inducer, further enhanced them [40, 45]. The consistent alterations observed across multiple biochemical, molecular, electron microscopy, and fluorescence assays provide strong evidence for the functional involvement of mitophagy in the protective effects of YHJF. This conclusion aligns with recent findings that enhancing PINK1/Parkin-mediated mitophagy alleviates inflammatory lung injury [2, 30]. Mechanistically, restored mitophagy was accompanied by reduced cytoplasmic mtDNA leakage and suppression of the cGAS–STING pathway, a canonical signalling axis linking mitochondrial danger signals to inflammatory activation [23, 24, 27, 34, 44]. Excessive release of mitochondrial DNA (mtDNA) is associated with severe inflammatory lung injury and poor prognosis of sepsis [9]. In our study, YHJF decreased the protein expression of cGAS and inhibited the phosphorylation of stimulator of STING, TBK1, and IRF3, and reduced downstream IFN-β, Ifi44, Cxcl10, and Isg15 expression. These findings suggest that YHJF suppresses mtDNA-driven inflammatory signaling by improving mitophagy, which is consistent with the reported effects of metformin [11]. Nevertheless, because we mainly assessed cGAS protein expression rather than its activation status, further studies are needed to determine whether YHJF directly modulates cGAS activation.

To determine whether macrophage-targeted protection could be translated into epithelial protection, we further established an MH-S and MLE-12 co-culture systemYHJF reduced IL-6, IL-1β, and TNF-α levels in the co-culture supernatant and attenuated LPS-induced apoptosis in MLE-12 cells. These findings indicate that YHJF-mediated restoration of macrophage mitochondrial homeostasis has functional consequences for neighbouring alveolar epithelial cells and suggest that macrophage-epithelial crosstalk contributes to its lung-protective effects.

The chemical profiling data further provide a preliminary material basis for the observed pharmacological effects. HPLC fingerprint validation confirmed good batch-to-batch stability of YHJF, and mass spectrometric analysis identified 101 compounds in the formula and 41 blood-entry components (SFig. 4, Table S5). Molecular docking showed that PINK1 exhibited favourable predicted binding affinities with several compounds, among which aloe-emodin, rhein, and genistein were notable. These findings are broadly consistent with previous studies showing that representative compounds from the component herbs of YHJF can regulate mitochondrial dysfunction, mitophagy, or cGAS–STING-related inflammatory signalling in sepsis-associated injury. For example, ginsenoside Rg1 alleviates SALI by reducing mitochondrial dysfunction [26], Ginsenoside Rg3 modulates the inflammatory response through the cGAS-STING pathway [43], emodin prevents sepsis by promoting mitophagy-related signaling [12], and notoginsenoside R1 ameliorates septic microvascular dysfunction by targeting mitochondrial imbalance [14]. Together, these findings support the multi-component and multi-target characteristics of YHJF, although the direct effects of these candidate monomers on macrophage mitophagy in SALI still require experimental validation.

Several limitations should be acknowledged. First, only female BALB/c mice were used, and potential sex-dependent differences in sepsis responses remain to be clarified. Second, although our proteomic, ultrastructural, molecular, and pharmacological data support the involvement of mitophagy, gene-targeted approaches are still needed for more rigorous mechanistic validation. Third, the co-culture model cannot fully recapitulate the in vivo alveolar microenvironment. Fourth, molecular docking was used only to prioritise candidate active constituents and does not demonstrate direct target engagement. Fifth, the in vitro experiments employed rat-derived YHJF-containing serum in mouse-derived cell lines; although this serum pharmacology strategy is widely used in TCM research, potential interspecies variability should be considered when interpreting the findings. Sixth, while mechanistic validation demonstrated robust effects at the cellular level, corresponding in vivo evidence remains limited. Finally, We recognize that pretreating mice with YHJF for 72 h before CLP, though helpful for ensuring adequate drug exposure in this initial study, represents a significant translational limitation. Clinical sepsis management requires post-onset intervention, whereas our protocol reflects a prophylactic paradigm. Future studies employing post-CLP administration at clinically relevant time points are essential to define the therapeutic window and validate YHJF efficacy in a treatment setting mirroring real-world practice.

## Conclusion

In conclusion, YHJF alleviates sepsis-associated lung injury by restoring mitochondrial homeostasis in alveolar macrophages. Its protective effects are linked to enhanced PINK1/Parkin-mediated mitophagy, reduced mtDNA leakage, suppression of cGAS-STING signalling, and macrophage-epithelial crosstalk. These findings support the therapeutic potential of YHJF in sepsis-associated acute lung injury.

## Supplementary Information

Supplementary Material 1. Figure 1: 7-Days survival rate in CLP mice (n=15). *P < 0.05 vs. CLP

Supplementary Material 2. Figure 2: CCK8 assay for MH-S cell viability: (A) LPS concentration test; *P < 0.05, **P < 0.01 vs. 0 ng group. (B) YHFJ medicated serum test; *P < 0.05, **P < 0.01 vs. LPS group

Supplementary Material 3. Figure 3: Proteomics data analysis of MH-S cells. (A) Principal component analysis plot of the proteomics data(CON group n=6; LPS group and YHJF group n=8). (B) Double volcano plot of differentially expressed proteins between the CON group and the LPS group, and between the LPS group and the YHJF group. (C) Venn diagram of shared DEPs across the three groups. (D) Reactome pathway enrichment analysis. (E) Biological Process analysis from Gene Ontology enrichment. (F) Molecular Function analysis from Gene Ontology enrichment. (G) Cellular Component analysis from Gene Ontology enrichment

Supplementary Material 4. Figure 4: Characterization of YHJF by HPLC and LC-MS. (A) HPLC fingerprint of YHJF extract and molecular structures of seven marker compounds; (B) TIC of YHJF in ESI⁺mode. (C) TIC of YHJF in ESI⁻ mode

Supplementary Material 5. Table S1. Primer pairs for genes

Supplementary Material 6. Table S2. 101 YHJF components in total

Supplementary Material 7. Table S3. 41 blood-entering compounds in YHJF

Supplementary Material 8. Table S4. Binding energy of the complex of key targets and compounds

Supplementary Material 9. Table S5. The fingerprint similarity value of YHJF

## Data Availability

The datasets used and/or analysed during the current study are available from the corresponding author on reasonable request.

## Abbreviations

ATP: Adenosine triphosphate
AMs: Alveolar macrophages
BALF: Bronchoalveolar lavage fluid
BID: Bis in die
BP: Biological processes
CLP: Cecal ligation and puncture
CCK-8: Cell Counting Kit-8
CC: Cellular components
DEX: Dexamethasone
DEPs: Differentially expressed proteins
ELISA: Enzyme-linked immunosorbent assay
FBS: Fetal bovine serum
FITC: Fluorescein isothiocyanate
GO: Gene ontology
GSEA: Gene set enrichment analysis
H&E: Hematoxylin and eosin
MF: Molecular functions
micro-CT: Micro computed tomography
Mdivi-1: Mitochondrial division inhibitor 1
mtDNA: Mitochondrial DNA
PCA: Principal component analysis
PE: Phycoerythrin
qRT-PCR: Quantitative real-time PCR
ROS: Reactive oxygen species
SALI: Sepsis-associated acute lung injury
TEM: Transmission electron microscopy
UA: Urolithin A
W/D: Wet-to-dry weight ratio
YHJF: Yiqi Huoxue Jiedu formula
