# Supplementary material for: Yiqi Huoxue Jiedu formula protects against sepsis-associated lung injury by modulating macrophage mitophagy and mtDNA-STING signaling
Source: Chin Med. 2026 Aug 4;21:207. doi: 10.1186/s13020-026-01475-0 (PMC13435792; doi:10.1186/s13020-026-01475-0)
Supplement: Supplementary file 1 [file 13020_2026_1475_MOESM5_ESM.docx]

**Table S1**  Primer pairs for genes

| Name | Fw Primer | Rw Primer |
| --- | --- | --- |
| LC3 | TGTGTCCACTCCCATCTCCGAAG | CCATTGCTGTCCCGAATGTCTCC |
| p62 | TGATTGAGTCCCTCTCCCAGATGC | CCGCTCCGATGTCATAGTTCTTGG |
| PINK1 | GCAGCAGTCAGCAGCCACTC | AGAGCCTCACACTCCAGGTTAGC |
| Parkin | GAGCACACCCAACCTCAGACAAG | ACGGAGGCACCAGATGACAGAG |
| STING | CCAAGAACCCACAGACGGAAACAG | GGAGGAGGTGCCACTGAGGTC |
| TBK1 | CAGTCTTCAGGACATCAGCAGCAG | CTGTCTCTTGGATGCGTGCCTTC |
| IRF3 | GAGGCTCGTGATGGTCAAGGTTG | AGTGGGTGGCTGTTGGAAATGTG |
| Isg15 | GCGAGCCTCTGAGCATCCTG | CCTTCTTCTTAAGCGTGTCTACAGTC |
| IFN-β | CTGGAGGAACTGGCAAAAGGATGG | GACGCTTATGTTGTTGCTGATGGC |
| Cxcl10 | GCCTCATCCTGCTGGGTCTG | TTCCCTATGGCCCTCATTCTCAC |
| Ifi44 | TAAGCAGTCAGAAGACAAGGCAATC | AGTCAGTAGTTTCCTCCAAGCAAAG |
| IL-6 | GACTTCCATCCAGTTGCCTTCTTG | GACAGGTCTGTTGGGAGTGGTATC |
| IL-1β | CTCGCAGCAGCACATCAACAAG | CCACGGGAAAGACACAGGTAGC |
| TNF-α | GTGCCTATGTCTCAGCCTCTTCTC | TGGTTTGTGAGTGTGAGGGTCTG |
| β-actin | CTCCATCCTGGCCTCGCTGT | GCTGCTACCTTCACCGTTCC |
