# Supplementary material for: Yiqi Huoxue Jiedu formula protects against sepsis-associated lung injury by modulating macrophage mitophagy and mtDNA-STING signaling
Source: Chin Med. 2026 Aug 4;21:207. doi: 10.1186/s13020-026-01475-0 (PMC13435792; doi:10.1186/s13020-026-01475-0)
Supplement: Supplementary file 2 [file 13020_2026_1475_MOESM6_ESM.docx]

**Table S2.** 101 YHJF components in total

| No. | tR/  min | m/z | Calculated mass | ppm | adduct | Formula | compound name |
| --- | --- | --- | --- | --- | --- | --- | --- |
| 1 | 0.98 | 177.05029 | 177.0506 | -1.74 | [M+H]+ | C_5_H_8_N_2_O_5_ | dencichine |
| 2 | 1.34 | 139.00238 | 139.00259 | -1.48 | [M+H-2H2O]+ | C_6_H_6_O_6_ | trans-Aconitic acid |
| 2 | 1.47 | 158.09224 | 158.0924 | -1.03 | [M+H-C12H25NO]+ | C_18_H_36_N_4_O_3_ | N-Lauroyl-L-arginine |
| 3 | 1.57 | 165.05441 | 165.05462 | -1.28 | [M+H]+ | C_9_H_8_O_3_ | p-Coumaric acid |
| 4 | 1.67 | 121.06492 | 121.06479 | 1.06 | [M+H]+ | C_8_H_8_O | Coumaran |
| 5 | 1.98 | 152.05647 | 152.05669 | -1.42 | [M+H-C8H12O4]+ | C_13_H_17_N_5_O_5_ | Isopropylideneguanosine |
| 6 | 2.40 | 151.07523 | 151.07536 | -0.83 | [M+H]+ | C_9_H_10_O_2_ | 2-Methoxy-4-vinylphenol |
| 7 | 2.65 | 195.11264 | 195.1128 | -0.84 | [M+H]+ | C_10_H_14_N_2_O_2_ | (5aR,10aR)-Octahydrodipyrrolo[1,2-a:1',2'-d]pyrazine-5,10-dione |
| 8 | 3.44 | 166.08599 | 166.08626 | -1.6 | [M+H]+ | C_9_H_11_NO_2_ | Phenylalanine |
| 9 | 4.05 | 339.06824 | 339.07106 | -8.31 | [M+H]+ | C_15_H_14_O_9_ | Quercetin Dihydrate |
| 10 | 6.61 | 188.0702 | 188.07061 | -2.15 | [M+H-H2O]+ | C_11_H_11_NO_3_ | 3-Indolyllactic acid |
| 11 | 7.69 | 579.14874 | 579.1497 | -1.66 | [M+H]+ | C_30_H_26_O_12_ | Procyanidin B2 |
| 12 | 8.38 | 291.08565 | 291.08631 | -2.28 | [M+H]+ | C_15_H_14_O_6_ | Epicatechin |
| 13 | 11.36 | 153.05437 | 153.05462 | -1.64 | [M+H]+ | C_8_H_8_O_3_ | Vanillin |
| 14 | 12.16 | 511.14172 | 511.13874 | 5.82 | [M+H]+ | C_30_H_22_O_8_ | (±)-Emodin bianthrone |
| 15 | 12.52 | 511.14185 | 511.13874 | 6.08 | [M+H]+ | C_30_H_22_O_8_ | Cis-Emodin bianthrone |
| 16 | 13.68 | 229.08527 | 229.08592 | -2.84 | [M+H]+ | C_14_H_12_O_3_ | Resveratrol |
| 17 | 14.28 | 377.14505 | 377.14556 | -1.35 | [M+H]+ | C_17_H_20_N_4_O_6_ | Vitamin B2 |
| 18 | 15.15 | 443.09778 | 443.09727 | 1.14 | [M+H]+ | C_22_H_18_O_10_ | Epicatechin gallate |
| 19 | 15.94 | 217.04933 | 217.04954 | -0.95 | [M+H-H2O]+ | C_12_H_10_O_5_ | 7-Hydroxy-4-methylcoumarin-3-acetic acid |
| 19 | 15.94 | 235.05983 | 235.0601 | -1.15 | [M+H]+ | C_12_H_10_O_5_ | Anhydrobrazilic acid |
| 20 | 17.78 | 293.10135 | 293.10196 | -2.1 | [M+H-C8H14O7]+ | C_23_H_30_O_13_ | 4-O-Acetyl-.beta.-D-fructofuranosyl 6-O-((2E)-3-phenylprop-2-enoyl)-.alpha.-D-glucopyranoside |
| 21 | 18.83 | 287.05453 | 287.05501 | -1.69 | [M+H-C6H10O4]+ | C_21_H_20_O_10_ | Afzelin |
| 22 | 19.08 | 469.07346 | 469.07562 | -4.61 | [M+K]+ | C_19_H_18_N_4_O_8_ | Bispyribac |
| 23 | 19.98 | 477.39331 | 477.39384 | -1.1 | [M+H]+ | C_30_H_52_O_4_ | Panaxatriol |
| 24 | 20.24 | 359.11212 | 359.11253 | -1.14 | [M+H]+ | C_19_H_18_O_7_ | Retusin |
| 25 | 20.89 | 191.06992 | 191.07027 | -1.84 | [M+H]+ | C_11_H_10_O_3_ | 4-Methylherniarin |
| 26 | 21.75 | 271.05975 | 271.0601 | -1.29 | [M+H]+ | C_15_H_10_O_5_ | Aloe emodin |
| 27 | 22.08 | 314.13805 | 314.13868 | -2.02 | [M+H]+ | C_18_H_19_NO_4_ | Moupinamide |
| 28 | 24.5 | 287.05402 | 287.05501 | -3.46 | [M+H]+ | C_15_H_10_O_6_ | Kaempferol |
| 29 | 25.92 | 247.09566 | 247.09649 | -3.34 | [M+H-C6H10O5]+ | C_20_H_24_O_9_ | Torachrysone 8-glucoside |
| 30 | 27.04 | 439.09937 | 439.09995 | -1.33 | [M+Na]+ | C_21_H_20_O_9_ | Chrysophanol 1-O-glucoside |
| 31 | 27.68 | 439.09909 | 439.09995 | -1.97 | [M+Na]+ | C_21_H_20_O_9_ | Chrysophanol 8-O-beta-D-glucoside |
| 31 | 27.73 | 405.35019 | 405.35158 | -3.42 | [M+H-C12H28O13]+ | C_42_H_72_O_13_ | Panaxoside Rg2 |
| 31 | 27.83 | 621.435 | 621.4361 | -1.76 | [M+H]+ | C_36_H_60_O_8_ | Ginsenoside Rk3 |
| 31 | 27.9 | 259.09604 | 259.09649 | -1.72 | [M+H]+ | C_15_H_14_O_4_ | Rhapontigenin |
| 31 | 27.93 | 421.14832 | 421.14931 | -2.35 | [M+H]+ | C_21_H_24_O_9_ | Rhapontigenin 3'-O-glucoside |
| 32 | 28.16 | 455.09424 | 455.09487 | -1.38 | [M+Na]+ | C_21_H_20_O_10_ | Aloe-emodin-8-O-β-D-glucopyranoside |
| 32 | 28.18 | 455.09427 | 455.09487 | -1.31 | [M+Na]+ | C_21_H_20_O_10_ | Emodin 8-glucoside |
| 33 | 28.83 | 477.39343 | 477.39384 | -0.85 | [M+H]+ | C_30_H_52_O_4_ | Protopanaxatriol |
| 34 | 28.94 | 405.15125 | 405.15439 | -7.76 | [M+H]+ | C_21_H_24_O_8_ | Desoxyrhaponticin |
| 35 | 29.12 | 423.36127 | 423.36214 | -2.06 | [M+H-C6H16O8]+ | C_36_H_62_O_9_ | Ginsenoside F1 |
| 35 | 29.15 | 203.1795 | 203.17943 | 0.36 | [M+H-C7H8O4]+ | C_22_H_30_O_4_ | Ferutinin |
| 35 | 29.16 | 441.37161 | 441.37271 | -2.49 | [M+H-C12H22O10]+ | C_42_H_70_O_12_ | Ginsenoside Rg4 |
| 35 | 29.22 | 823.4798 | 823.48143 | -1.98 | [M+Na]+ | C_42_H_72_O_14_ | Ginsenoside Rg1 |
| 36 | 29.69 | 909.4801 | 909.48182 | -1.89 | [M+Na]+ | C_45_H_74_O_17_ | Asparanin B |
| 37 | 30.64 | 243.10114 | 243.10157 | -1.77 | [M+H]+ | C_15_H_14_O_3_ | 4'-Methoxyresveratrol |
| 38 | 31.46 | 271.0596 | 271.0601 | -1.84 | [M+H]+ | C_15_H_10_O_5_ | Emodin |
| 39 | 33.7 | 645.4328 | 645.43369 | -1.38 | [M+Na]+ | C_36_H_62_O_8_ | 20(R)-Ginsenoside Rh2 |
| 40 | 35.14 | 807.4845 | 807.48651 | -2.49 | [M+H-3H2O]+ | C_42_H_72_O_13_ | 20(R)-Ginsenoside Rg2 |
| 41 | 35.32 | 661.42725 | 661.4286 | -2.05 | [M+Na]+ | C_36_H_62_O_9_ | (20R)-Ginsenoside Rh1 |
| 42 | 36.04 | 661.42743 | 661.4286 | -1.78 | [M+Na]+ | C_36_H_62_O_9_ | Ginsenoside Rh1 |
| 43 | 37.99 | 1263.63232 | 1263.63442 | -1.66 | [M+Na]+ | C_59_H_100_O_27_ | Notoginsenoside Fa |
| 44 | 38.17 | 439.35641 | 439.35706 | -1.47 | [M+H-H2O]+ | C_30_H_48_O_3_ | Ursolic acid |
| 45 | 38.73 | 969.53705 | 969.53934 | -2.36 | [M+Na]+ | C_48_H_82_O_18_ | Gypenoside XVII |
| 45 | 38.76 | 407.36603 | 407.36723 | -2.94 | [M+H-3H2O]+ | C_30_H_52_O_3_ | Protopanaxadiol |
| 45 | 38.79 | 1109.60767 | 1109.61022 | -2.29 | [M+H]+ | C_54_H_92_O_23_ | Ginsenoside Rb1 |
| 46 | 39.18 | 1211.6499 | 1211.64191 | 6.6 | [M+H]+ | C_58_H_98_O_26_ | Notoginsenoside Fc |
| 47 | 39.94 | 149.1322 | 149.13248 | -1.86 | [M+H-C4H10O]+ | C_15_H_26_O | (cis+trans)-Nerodilol |
| 47 | 39.94 | 1079.59729 | 1079.59965 | -2.19 | [M+H]+ | C_53_H_90_O_22_ | Ginsenoside Rc |
| 48 | 40.15 | 1079.59729 | 1079.59965 | -2.19 | [M+H]+ | C_53_H_90_O_22_ | Ginsenoside Rb2 |
| 49 | 40.85 | 205.19474 | 205.19508 | -1.64 | [M+H]+ | C_15_H_24_ | β-Caryophyllene |
| 50 | 41.41 | 749.48114 | 749.48344 | -3.07 | [M+H-2H2O]+ | C_42_H_72_O_13_ | Ginsenoside Rg3 |
| 51 | 41.76 | 789.47424 | 789.47595 | -2.16 | [M+Na]+ | C_42_H_70_O_12_ | Ginsenoside Rk1 |
| 51 | 41.76 | 645.4328 | 645.43369 | -1.38 | [M+Na]+ | C_36_H_62_O_8_ | Ginsenoside Rh2 |
| 52 | 41.94 | 645.42999 | 645.43369 | -5.73 | [M+Na]+ | C_36_H_62_O_8_ | Ginsenoside C-K |
| 53 | 42.33 | 605.44012 | 605.44118 | -1.75 | [M+Na]+ | C_36_H_60_O_7_ | Ginsenoside Rh3 |
| 54 | 43.88 | 338.34091 | 338.34174 | -2.46 | [M+H]+ | C_22_H_43_NO | Erucamide |
| 55 | 0.96 | 191.05527 | 191.05501 | 1.34 | [M-H-C9H6O3]- | C_16_H_18_O_9_ | Neochlorogenic acid |
| 55 | 0.99 | 387.11486 | 387.11332 | 3.99 | [M+HCOO]- | C_12_H_22_O_11_ | Sucrose |
| 55 | 0.99 | 387.11475 | 387.11332 | 3.7 | [M+HCOO]- | C_12_H_22_O_11_ | D-(+)-Trehalose |
| 56 | 1.39 | 173.00819 | 173.00806 | 0.73 | [M-H-H2O]- | C_6_H_8_O_7_ | Citric acid |
| 57 | 1.99 | 169.0132 | 169.01315 | 0.3 | [M-H-C15H12O6]- | C_22_H_18_O_11_ | (-)-Gallocatechin 3-gallate |
| 57 | 1.99 | 125.02305 | 125.02332 | -2.16 | [M-H]- | C_6_H_6_O_3_ | Maltol |
| 58 | 7.71 | 577.13574 | 577.13405 | 2.92 | [M-H]- | C_30_H_26_O_12_ | Procyanidin B2 |
| 59 | 8.43 | 579.15147 | 579.1497 | 3.05 | [2M-H]- | C_15_H_14_O_6_ | (-)-Catechin |
| 60 | 11.99 | 335.07751 | 335.07614 | 4.08 | [M+HCOO]- | C_15_H_14_O_6_ | Catechin |
| 61 | 17.51 | 499.0864 | 499.0871 | -1.41 | [M-H]- | C_24_H_20_O_12_ | 6,7'-Dihydroxy-2,2'-dioxo-2H,2'H-[8,8'-bichromen]-7-yl hexopyranoside |
| 62 | 17.62 | 609.14728 | 609.14501 | 3.72 | [M-H]- | C_27_H_30_O_16_ | Kaempferol 3-o-sophoroside |
| 63 | 18.92 | 463.08914 | 463.0871 | 4.41 | [M-H]- | C_21_H_20_O_12_ | Quercetin-3-o-glucoside |
| 64 | 19.10 | 445.078 | 445.07654 | 3.29 | [M-H]- | C_21_H_18_O_11_ | Rhein 8-Glucoside |
| 64 | 19.10 | 541.1355 | 541.13405 | 2.67 | [M-H]- | C_27_H_26_O_12_ | Resveratrol 4'-(6-galloylglucoside) |
| 65 | 19.46 | 609.14709 | 609.14501 | 3.41 | [M-H]- | C_27_H_30_O_16_ | Rutin |
| 66 | 20.11 | 447.09399 | 447.09219 | 4.03 | [M-H]- | C_21_H_20_O_11_ | Kaempferol-3-o-glucoside |
| 67 | 22.60 | 861.18973 | 861.18727 | 2.86 | [M-H]- | C_42_H_38_O_20_ | Sennoside A |
| 68 | 23.83 | 461.10974 | 461.10784 | 4.13 | [M-H]- | C_22_H_22_O_11_ | .beta.-D-Glucopyranose, 6-[(2E)-3-phenyl-2-propenoate] 1-(3,4,5-trihydroxybenzoate) |
| 69 | 24.12 | 301.03571 | 301.03428 | 4.75 | [M-H]- | C_15_H_10_O_7_ | Quercetin |
| 70 | 25.85 | 389.12482 | 389.12309 | 4.44 | [M-H]- | C_20_H_22_O_8_ | Polydatin |
| 71 | 27.05 | 415.10387 | 415.10236 | 3.64 | [M-H]- | C_21_H_20_O_9_ | Chrysophanol 8-O-beta-D-glucoside |
| 72 | 27.56 | 577.13599 | 577.13405 | 3.36 | [M-H]- | C_30_H_26_O_12_ | Procyanidin B3 |
| 72 | 27.67 | 415.10382 | 415.10236 | 3.52 | [M-H]- | C_21_H_20_O_9_ | Chrysophanein |
| 72 | 27.67 | 253.0506 | 253.04954 | 4.21 | [M-H]- | C_15_H_10_O_4_ | Chrysophanol |
| 73 | 27.81 | 931.52631 | 931.52609 | 0.23 | [M-H]- | C_47_H_80_O_18_ | Sanchinoside R1 |
| 73 | 27.91 | 431.09845 | 431.09727 | 2.73 | [M-H]- | C_21_H_20_O_10_ | Vitexin |
| 73 | 27.91 | 257.082 | 257.08084 | 4.53 | [M-H]- | C_15_H_14_O_4_ | Isorhapontigenin |
| 74 | 28.16 | 431.09851 | 431.09727 | 2.87 | [M-H]- | C_21_H_20_O_10_ | Emodin 8-glucoside |
| 75 | 28.89 | 925.48181 | 925.47914 | 2.88 | [M-H]- | C_47_H_74_O_18_ | Chikusetsusaponin IV |
| 76 | 29.03 | 991.54874 | 991.54722 | 1.53 | [M+HCOO]- | C_48_H_82_O_18_ | Ginsenoside Re |
| 76 | 29.13 | 845.49105 | 845.48931 | 2.06 | [M+HCOO]- | C_42_H_72_O_14_ | Ginsenoside Rg1 |
| 76 | 29.20 | 845.49152 | 845.48931 | 2.61 | [M+HCOO]- | C_42_H_72_O_14_ | Pseudoginsenoside F11 |
| 77 | 29.67 | 885.48651 | 885.48423 | 2.58 | [M-H]- | C_45_H_74_O_17_ | Asparanin B |
| 78 | 30.17 | 285.04071 | 285.03936 | 4.72 | [M-H]- | C_15_H_10_O_6_ | Citreorosein |
| 79 | 31.11 | 283.0614 | 283.0601 | 4.59 | [M-H]- | C_16_H_12_O_5_ | Parietin |
| 79 | 31.11 | 445.11441 | 445.11292 | 3.34 | [M-H]- | C_22_H_22_O_10_ | Physcion 8-O-β-D-glucopyranoside |
| 80 | 31.37 | 815.48107 | 815.47875 | 2.85 | [M+FA-H]- | C_41_H_70_O_13_ | Ginsenoside F3 |
| 81 | 32.2 | 297.04068 | 297.03936 | 4.43 | [M-H]- | C_16_H_10_O_6_ | 3,8-Dihydroxy-1-methylanthraquinone-2-carboxylic acid |
| 82 | 32.74 | 815.48108 | 815.47875 | 2.86 | [M+HCOO]- | C_41_H_70_O_13_ | Ginsenoside F5 |
| 83 | 33.79 | 845.4913 | 845.48931 | 2.35 | [M+HCOO]- | C_42_H_72_O_14_ | Ginsenoside Rf |
| 84 | 34.22 | 769.47388 | 769.47327 | 0.79 | [M-H]- | C_41_H_70_O_13_ | 20(r)-notoginsenoside R2 |
| 85 | 34.76 | 283.02496 | 283.02371 | 4.4 | [M-H]- | C_15_H_8_O_6_ | Rhein |
| 85 | 34.76 | 239.03484 | 239.03389 | 3.99 | [M-H]- | C_14_H_8_O_4_ | Danthron |
| 86 | 35.12 | 829.49664 | 829.4944 | 2.7 | [M+HCOO]- | C_42_H_72_O_13_ | Ginsenoside F2 |
| 87 | 35.3 | 805.45227 | 805.44995 | 2.89 | [M+Cl]- | C_41_H_70_O_13_ | Notoginsenoside R2 |
| 88 | 35.77 | 829.49701 | 829.4944 | 3.15 | [M+HCOO]- | C_42_H_72_O_13_ | 20(S)-Ginsenoside Rg3 |
| 89 | 38.05 | 955.49123 | 955.48971 | 1.59 | [M-H]- | C_48_H_76_O_19_ | Chikusetsusaponin V |
| 90 | 38.15 | 955.49133 | 955.48971 | 1.7 | [M-H]- | C_48_H_76_O_19_ | Chikusetsusaponin V |
| 91 | 38.78 | 1153.60208 | 1153.60004 | 1.76 | [M+FA-H]- | C_54_H_92_O_23_ | Ginsenoside Rb1 |
| 92 | 38.92 | 925.48169 | 925.47914 | 2.75 | [M-H]- | C_47_H_74_O_18_ | Pseudoginsenoside RT1 |
| 93 | 39.13 | 1123.59096 | 1123.58948 | 1.32 | [M+FA-H]- | C_53_H_90_O_22_ | Ginsenoside Rb3 |
| 93 | 39.16 | 1209.62793 | 1209.62626 | 1.38 | [M-H]- | C_58_H_98_O_26_ | Notoginsenoside FP2 |
| 94 | 39.63 | 269.04578 | 269.04445 | 4.94 | [M-H]- | C_37_H_38_N_2_O_6_ | Cepharanthine |
| 95 | 40.67 | 269.04581 | 269.04445 | 5.06 | [M-H-C6H10O4]- | C_21_H_20_O_9_ | Frangulin A |
| 96 | 40.74 | 811.48639 | 811.48383 | 3.15 | [M+FA-H]- | C_42_H_70_O_12_ | Ginsenoside Rg5 |
| 97 | 40.84 | 991.54915 | 991.54722 | 1.95 | [M+HCOO]- | C_48_H_82_O_18_ | Ginsenoside Rd |
| 98 | 41.15 | 961.53937 | 961.53666 | 2.82 | [M+HCOO]- | C_47_H_80_O_17_ | Notoginsenoside Ft1 |
| 99 | 41.43 | 829.4967 | 829.4944 | 2.78 | [M+HCOO]- | C_42_H_72_O_13_ | Panaxoside Rg2 |
| 99 | 41.43 | 829.4967 | 829.4944 | 2.78 | [M+HCOO]- | C_42_H_72_O_13_ | 20(R)-Ginsenoside Rg2 |
| 100 | 41.78 | 765.47949 | 765.47835 | 1.48 | [M-H]- | C_42_H_70_O_12_ | Ginsenoside Rg6 |
| 101 | 42.83 | 255.23299 | 255.23186 | 4.44 | [M-H]- | C_16_H_32_O_2_ | Methyl pentadecanoate |
