# Supplementary material for: Yiqi Huoxue Jiedu formula protects against sepsis-associated lung injury by modulating macrophage mitophagy and mtDNA-STING signaling
Source: Chin Med. 2026 Aug 4;21:207. doi: 10.1186/s13020-026-01475-0 (PMC13435792; doi:10.1186/s13020-026-01475-0)
Supplement: Supplementary file 3 [file 13020_2026_1475_MOESM7_ESM.docx]

**Table S3**. 41 blood-entering compounds in YHJF

| No. | tR/min | m/z | Adduct | Formula | Compound name |
| --- | --- | --- | --- | --- | --- |
| 1 | 1.47 | 158.09224 | [M+H-C_12_H_25_NO]+ | C_18_H_36_N_4_O_3_ | N-Lauroyl-L-arginine |
| 2 | 13.68 | 229.08527 | [M+H]+ | C_14_H_12_O_3_ | Resveratrol |
| 3 | 15.94 | 217.04933 | [M+H-H_2_O]+ | C_12_H_10_O_5_ | 7-Hydroxy-4-methylcoumarin-3-acetic acid |
| 4 | 15.94 | 235.05983 | [M+H]+ | C_12_H_10_O_5_ | Anhydrobrazilic acid |
| 5 | 20.89 | 191.06992 | [M+H]+ | C_11_H_10_O_3_ | 4-Methylherniarin |
| 6 | 27.04 | 439.09937 | [M+Na]+ | C_21_H_20_O_9_ | Chrysophanol 1-O-glucoside |
| 7 | 27.68 | 439.09909 | [M+Na]+ | C_21_H_20_O_9_ | Chrysophanol 8-O-beta-D-glucoside |
| 8 | 27.73 | 405.35019 | [M+H-C_12_H_28_O_13_]+ | C_42_H_72_O_13_ | Panaxoside Rg2 |
| 9 | 28.94 | 405.15125 | [M+H]+ | C_21_H_24_O_8_ | Desoxyrhaponticin |
| 10 | 29.12 | 423.36127 | [M+H-C_6_H_16_O_8_]+ | C_36_H_62_O_9_ | Ginsenoside F1 |
| 11 | 29.15 | 203.1795 | [M+H-C_7_H_8_O_4_]+ | C_22_H_30_O_4_ | Ferutinin |
| 12 | 29.16 | 441.37161 | [M+H-C_12_H_22_O_10_]+ | C_42_H_70_O_12_ | Ginsenoside Rg4 |
| 13 | 29.22 | 823.4798 | [M+Na+ | C_42_H_72_O_14_ | Ginsenoside Rg1 |
| 14 | 30.64 | 243.10114 | [M+H]+ | C_15_H_14_O_3_ | 4'-Methoxyresveratrol |
| 15 | 31.46 | 271.0596 | [M+H]+ | C_15_H_10_O_5_ | Emodin |
| 16 | 35.32 | 661.42725 | [M+Na]+ | C_36_H_62_O_9_ | (20R)-Ginsenoside Rh1 |
| 17 | 36.04 | 661.42743 | [M+Na]+ | C_36_H_62_O_9_ | Ginsenoside Rh1 |
| 18 | 38.73 | 969.53705 | [M+Na]+ | C_48_H_82_O_18_ | Gypenoside XVII |
| 19 | 38.76 | 407.36603 | [M+H-_3_H_2_O]+ | C_30_H_52_O_3_ | Protopanaxadiol |
| 20 | 42.33 | 605.44012 | [M+Na]+ | C_36_H_60_O_7_ | Ginsenoside Rh3 |
| 21 | 0.96 | 191.05527 | [M-H-C_9_H_6_O_3_]- | C_16_H_18_O_9_ | Neochlorogenic acid |
| 22 | 25.85 | 389.12482 | [M-H]- | C_20_H_22_O_8_ | Polydatin |
| 23 | 27.56 | 577.13599 | [M-H]- | C_30_H_26_O_12_ | Procyanidin B3 |
| 24 | 27.67 | 415.10382 | [M-H]- | C_21_H_20_O_9_ | Chrysophanein |
| 25 | 27.67 | 253.0506 | [M-H]- | C_15_H_10_O_4_ | Chrysophanol |
| 26 | 27.81 | 931.52631 | [M-H]- | C_47_H_80_O_18_ | Sanchinoside R1 |
| 27 | 27.91 | 257.082 | [M-H]- | C_15_H_14_O_4_ | Isorhapontigenin |
| 28 | 29.03 | 991.54874 | [M+HCOO]- | C_48_H_82_O_18_ | Ginsenoside Re |
| 29 | 29.20 | 845.49152 | [M+HCOO]- | C_42_H_72_O_14_ | Pseudoginsenoside F11 |
| 30 | 32.20 | 297.04068 | [M-H]- | C_16_H_10_O_6_ | 3,8-Dihydroxy-1-methylanthraquinone-2-carboxylic acid |
| 31 | 34.76 | 283.02496 | [M-H]- | C_15_H_8_O_6_ | Rhein |
| 32 | 34.76 | 239.03484 | [M-H]- | C_14_H_8_O_4_ | Danthron |
| 33 | 35.12 | 829.49664 | [M+HCOO]- | C_42_H_72_O_13_ | Ginsenoside F2 |
| 34 | 35.30 | 805.45227 | [M+Cl]- | C_41_H_70_O_13_ | Notoginsenoside R2 |
| 35 | 35.77 | 829.49701 | [M+HCOO]- | C_42_H_72_O_13_ | 20(S)-Ginsenoside Rg3 |
| 36 | 38.05 | 955.49123 | [M-H]- | C_48_H_76_O_19_ | Chikusetsusaponin V |
| 37 | 38.78 | 1153.60208 | [M+HCOO]- | C_54_H_92_O_23_ | Ginsenoside Rb1 |
| 38 | 39.13 | 1123.59096 | [M+HCOO]- | C_54_H_92_O_23_ | Ginsenoside Rb3 |
| 39 | 39.16 | 1209.62793 | [M-H]- | C_58_H_98_O_26_ | Notoginsenoside FP2 |
| 40 | 39.63 | 269.04578 | [M-H]- | C_37_H_38_N_2_O_6_ | Cepharanthine |
| 41 | 40.84 | 991.54915 | [M+HCOO]- | C_48_H_82_O_18_ | Ginsenoside Rd |
