# Supplementary material for: Yiqi Huoxue Jiedu formula protects against sepsis-associated lung injury by modulating macrophage mitophagy and mtDNA-STING signaling
Source: Chin Med. 2026 Aug 4;21:207. doi: 10.1186/s13020-026-01475-0 (PMC13435792; doi:10.1186/s13020-026-01475-0)
Supplement: Supplementary file 4 [file 13020_2026_1475_MOESM8_ESM.docx]

**Table S4.** Binding energy of the complex of key targets and compounds

| Compounds | PINK1 | Parkin | LC3 | p62 |
| --- | --- | --- | --- | --- |
| (-)-Epigallocatechin | -9.5 | -8.2 | -8.4 | -7.5 |
| Aloeemodin | -10.3 | -8.6 | -7.2 | -7.2 |
| Genistein | -9.9 | -8.3 | -6.2 | -6.9 |
| GinsenosideRg1 | -9.1 | -8.1 | -10.6 | -7.4 |
| GinsenosideRg3 | -8.2 | -7.9 | -5.1 | -5.9 |
| Methyl4-coumarate | -8.3 | -7 | -5.8 | -5.8 |
| Rhein | -10.6 | -8.9 | -7.2 | -7.6 |
| Ursolicacid | -8.6 | -8.8 | -8 | -7.9 |
| Apigenin 7-glucuronide | -11.4 | -9.3 | -4.8 | -7.5 |
| Chlorogenate | -9.9 | -8.5 | -6.5 | -7.4 |
| Chrysophanol | -10.3 | -8.8 | -7.3 | -7.3 |
| CinnamoylglycineN | -9.2 | -7.6 | -6.2 | -6.4 |
| Citreorosein | -10.3 | -8.5 | -6.8 | -7.2 |
| Coumaric acid 4-O-glucoside | -9.7 | -8.1 | -4.5 | -7 |
| Cryptochlorogenic acid | -10 | -8.4 | -5.8 | -7.3 |
| Dihydrokaempferol | -9.9 | -8 | -6.6 | -6.9 |
| Emodin 8-glucoside | -11.3 | -9 | -5.3 | -7.3 |
| Emodol | -10.5 | -8.7 | -7.2 | -7.3 |
| Eriodictyol-7-O-glucoside | -10.9 | -9.2 | -5.6 | -7.6 |
| Kaempferol 3-glucuronoside | -9.9 | -8.8 | -4.9 | -6.9 |
| Luteolin 7-glucuronide | -11.3 | -9.2 | -5.2 | -7.6 |
| Methyl 4-coumarate4- | -8.3 | -7 | -5.8 | -5.8 |
| Methyl beta-D-glucopyranoside | -7 | -6.8 | -4.7 | -6.2 |
| Parietin | -10.3 | -8.4 | -7 | -7.2 |
| Protopanaxadiol (20R)- | -9.3 | -8.8 | -3 | -7.3 |
| Quercetin-3-o-glucoside | -11.2 | -8.8 | -5.4 | -7.2 |
