# Supplementary material for: Yiqi Huoxue Jiedu formula protects against sepsis-associated lung injury by modulating macrophage mitophagy and mtDNA-STING signaling
Source: Chin Med. 2026 Aug 4;21:207. doi: 10.1186/s13020-026-01475-0 (PMC13435792; doi:10.1186/s13020-026-01475-0)
Supplement: Supplementary file 5 [file 13020_2026_1475_MOESM9_ESM.docx]

**Table S5.** The fingerprint similarity value of YHJF

|  | S1 | S2 | S3 | S4 | S5 | S6 | R |
| --- | --- | --- | --- | --- | --- | --- | --- |
| S1 | 1.000 | 0.989 | 0.984 | 0.989 | 0.989 | 0.995 | 0.989 |
| S2 | 0.989 | 1.000 | 0.983 | 0.997 | 0.999 | 0.993 | 0.998 |
| S3 | 0.984 | 0.983 | 1.000 | 0.982 | 0.987 | 0.990 | 0.978 |
| S4 | 0.989 | 0.997 | 0.982 | 1.000 | 0.993 | 0.987 | 0.997 |
| S5 | 0.989 | 0.999 | 0.987 | 0.993 | 1.000 | 0.989 | 0.997 |
| S6 | 0.995 | 0.993 | 0.990 | 0.987 | 0.989 | 1.000 | 0.992 |
| R | 0.989 | 0.998 | 0.978 | 0.997 | 0.997 | 0.992 | 1.000 |
